# Pitfalls in Interpreting mp-MRI of the Prostate: A Pictorial Review with Pathologic Correlation

**DOI:** 10.1007/s13244-015-0426-9

**Published:** 2015-09-18

**Authors:** V. Panebianco, F. Barchetti, J. Barentsz, A. Ciardi, F. Cornud, J. Futterer, G. Villeirs

**Affiliations:** Department of Radiological Sciences, Oncology & Pathology- Sapienza Unviersity of Rome, V.le Regina Elena, 324 00161 Roma, Italy; Department of Radiology and Nuclear Medicine, Radboud University Medical Centre, Nijmegen, The Netherlands; Department of Radiology, Hôpital Cochin, Paris Descartes University, Sorbonne Paris Cité, Paris, France; Department of Radiology, Ghent University Hospital, Ghent, Belgium

**Keywords:** Prostate, mp-MRI, Pitfalls, Prostate MRI, Differential diagnosis

## Abstract

**Objectives:**

The purpose of this pictorial review is to present a wide spectrum of prostate multiparametric MRI (mp-MRI) pitfalls that may occur in clinical practice, with radiological and pathological correlation.

**Methods:**

All examinations were performed according to ESUR Guidelines protocols.

**Results and Conclusion:**

mp-MRI imaging of the prostate often leads to interpreting doubts and misdiagnosis due to the many interpretative pitfalls that a tissue, whether healthy or treated, may cause. These “false-positive” findings may occur in each stage of the disease history, from the primary diagnosis and staging, to the post-treatment stage, and whether they are caused by the tissue itself or are iatrogenic, their recognition is critical for proper treatment and management. Knowledge of these known pitfalls and their interpretation in the anatomical-radiological context can help radiologists avoid misdiagnosis and consequently mistreatment.

***Main Messages*:**

• *Some physiological changes in the peripheral and central zone may simulate prostate cancer*.

• *Technical errors*, *such as mispositioned endorectal coils*, *can affect the mp-MRI interpretation*.

• *Physiological changes post*-*treatment can simulate recurrence*

## Introduction

In the past two decades, technological innovations related to scientific research in the diagnosis of prostate cancer (PCa) have allowed magnetic resonance imaging (MRI) to become the current main imaging modality for the detection, localization, staging, grading and response assessment to therapy [1–4]. Great interest has been shown in multiparametric MRI (mp-MRI), which combines anatomic T2-weighted (T2W) imaging with functional techniques. The detection of PCa on T2W can be confounded by false-positive findings such as prostatitis, post-biopsy haemorrhage, benign prostatic hyperplasia, fibrosis, radiation and hormonal tissue changes [5, 6]. In order to improve the diagnostic accuracy of PCa imaging, functional MR imaging techniques have been applied, such as Diffusion-Weighted MR Imaging (DWI) [7–9], proton (1H) MR Spectroscopic Imaging (MRSI) [10–12], and Dynamic Contrast-Enhanced MR Imaging (DCE-MRI) [13]. The combination of anatomic, biological and metabolic information offered by multiparametric MRI (mp-MRI) provides a promising imaging tool for improving many aspects of PCa management. Moreover, mp-MRI has recently also been proposed as a tool that is more useful than other imaging procedures for the diagnosis of local recurrence of PCa after radiation treatment or radical prostatectomy [14–18]. In order to allow for a standardized interpretation, and to objectively visualize the contribution of mp-MRI to predict the presence of significant cancer, a scoring system similar to that employed successfully by breast radiologists (BI-RADS) [19], has been designed for mp-MRI (PI-RADS) [20]. In this scoring system, every imaging technique (T2W, DW, DCE-MRI and MRSI) is scored on a five-point scale, based on the probability that a combination of mp-MRI findings on T2w, DWI and DCE findings correlates with the presence of a clinically significant cancer on each portion of the gland, as recently illustrated on the PI-RADS v2. Because of multiple diagnostic misunderstandings that may arise from the individual parameters forming the study protocol, the system of “score” with PI-RADS was introduced. Each value assigned to each sequence is weighted in relation to the sequence considered to be “dominant” for each zone of the gland; the DWI is considered the main sequence for the peripheral zone (PZ), while the T2 is the main sequence for the transition zone (TZ). An accurate assessment of an mp-MRI, in addition to the PI-RADS score, requires a comprehensive evaluation of the gland, including unavoidable knowledge of anatomy, pathology and clinical data in order to avoid certain diagnostic pitfalls, both false-positive and false-negative interpretations, that prostate MRI can show [21]. In this paper, we present a collection of pitfalls of interpreting prostate mp-MRI and propose a series of solutions for difficult cases (Tables 1 and 2).

**Table 1:** Pitfalls classification based on categories before treatment

Category	Pitfall
1. Pitfalls in primary diagnosis	1. Bilateral basal hypointense zones (moustache sign)
	2. Median posterior hypointense area at the middle third of the gland
	3. Transition zone prostate cancer versus BPH foci of stromal hyperplasia
	4. Ectopic BPH nodule
	5. Granulomatous prostatitis after intravesical instillation of BCG
	6. Hypertrophic anterior fibromuscular stroma
	7. Periprostatic venous plexus and neurovascular bundle
2. Pitfall in the staging	1. T3 versus T2 (overlapping with pitfalls in primary diagnosis: granulomatous prostatitis, periprostatic venous plexus and neurovascular bundle)
	5. Bone findings
	6. Controversial on lymph nodes
3. Artifacts/iatrogenic changes	1. Mispositioned endorectal coil
	2. Post-biopsy changes
	3. Lymphoceles

**Table 2:** Pitfalls classification based on categories after treatment

Category	Pitfall
4. Pitfalls after surgical treatment	1. Residual glandular tissue
	2. Fibrosis
	3. Retained seminal vesicles
	4. Sealed off veins
	5. Prominent periprostatic venous plexus
	6. Residual verumontanum
5. Pitfalls after radiation/hormone deprivation therapy and after focal therapies	1. RT-induced capsular irregularity may hinder evaluation of extracapsular extension
	2. Focal regions of T2-hypointensity may represent treated tumour and not local recurrence
	3. Focal therapies-induced enhancing of reactive prostate tissue may hamper the assessment of persistent/residual disease

## Pitfalls in Primary Diagnosis

### Bilateral Basal Hypointense Zones (moustache sign)

**Fig. 1:**
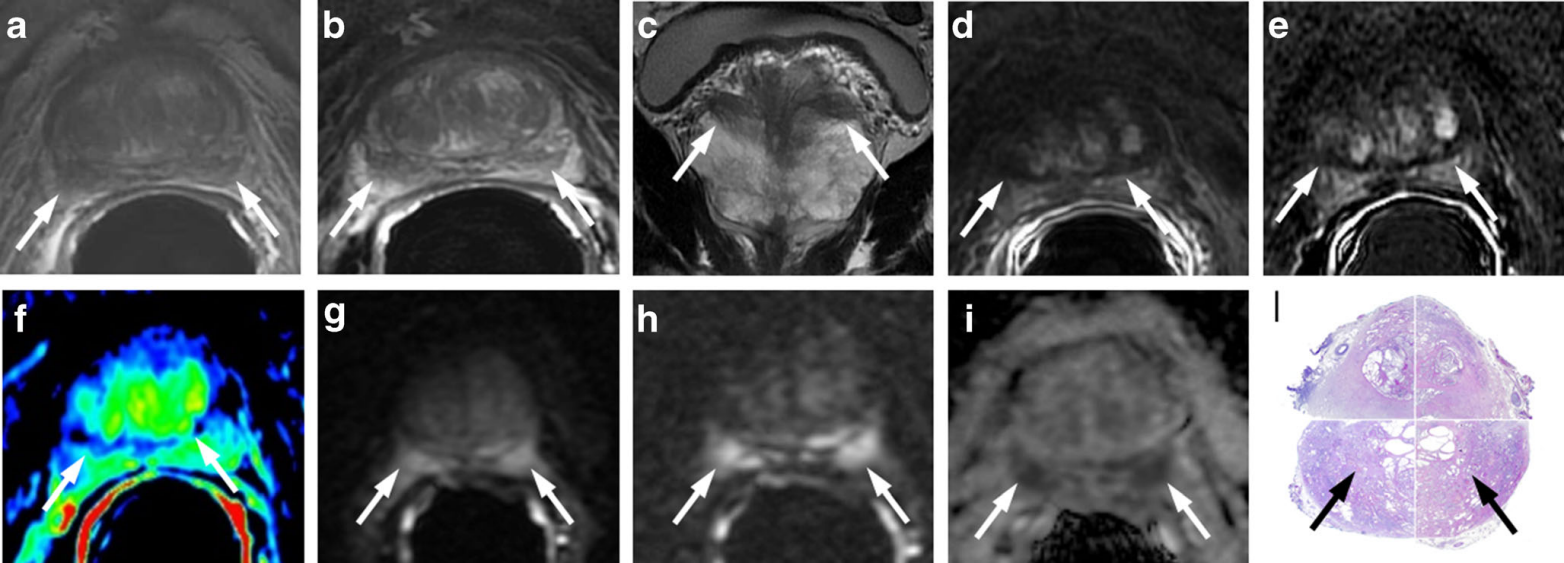
A 74-year-old man with PCa at the apex and bilateral basal hypointense nodular zones (moustache sign) (*white arrows*). **a** Axial T2-weighted fast spin-echo image (4564/110). **b** axial T2-weighted fat saturated fast spin-echo image (4941/116) and **c** coronal T2-weighted fast spin-echo image (5059/120) showing symmetric, bilateral, well-defined homogeneous hypointense zones at the base of the prostate gland. **d** Axial perfusion gradient-echo T1-weighted, **e** gradient-echo T1-weighted subtracted image and **f** colour DCE (dynamic contrast enhance) MR map displaying mild enhancement of the hypointense zones. **g** Axial DWI image with b value = 1000, **h** 3000 mm^2^/sec, and **i** ADC map showing restricted diffusion phenomena of water molecules in the hypointense basal sites detected on T2-weighted images. According to the PIRADSv2 scoring system, the score for T2, DCE and DWI is, respectively: 3, + and 4; the overall score is 4, which means highly clinically significant cancer is likely to be present. **i** Photomicrograph from a prostatectomy specimen shows the compression exerted by BPH on the central zone, bilaterally (*black arrows*)

It is very common to find at the base of the prostate gland bilateral and usually symmetric areas (moustache-like) of homogeneous low signal intensity areas on T2-weighted morphologic images located on either side of the ejaculatory ducts. These zones also show restricted diffusion with low ADC values on the ADC maps, sometimes symmetric contrast enhancement on perfusion sequences and increased choline peaks on MRSI [22]. All these malignant-looking morphologic and functional features can erroneously lead to the diagnosis of bilateral PCa. These symmetrical areas are due to compression of the central zone by benign prostatic hyperplasia (BPH) against the transition zone (Fig. 1) These typical oval-shaped areas usually appear asymmetric between them, and when this happens, the coronal T2-weighted sequence is the most accurate to settle the differential diagnosis. Morphological aspects of the moustache sign include symmetrical oval shape, with sharp margins and homogeneous dark signal intensity (SI), where foci of PCa generally have more heterogeneous appearance with ill-defined margins. Sometimes a similar appearance may be due by an ectopic stromal BPH nodule, whereas an adenomatous BPH ectopic nodule or a cystic atrophy can be easily recognized by the hyperintense SI. Compressed central zone usually does not show enhancement, whereas protrusion of BPH nodules does. The use of sagittal and/or coronal images is crucial for this differentiation, as compression of the central zone is never continuous to PCa arising in the PZ (Fig. 1c).

### Median Posterior Hypointense Area at the Middle Third of the Gland

**Fig. 2:**
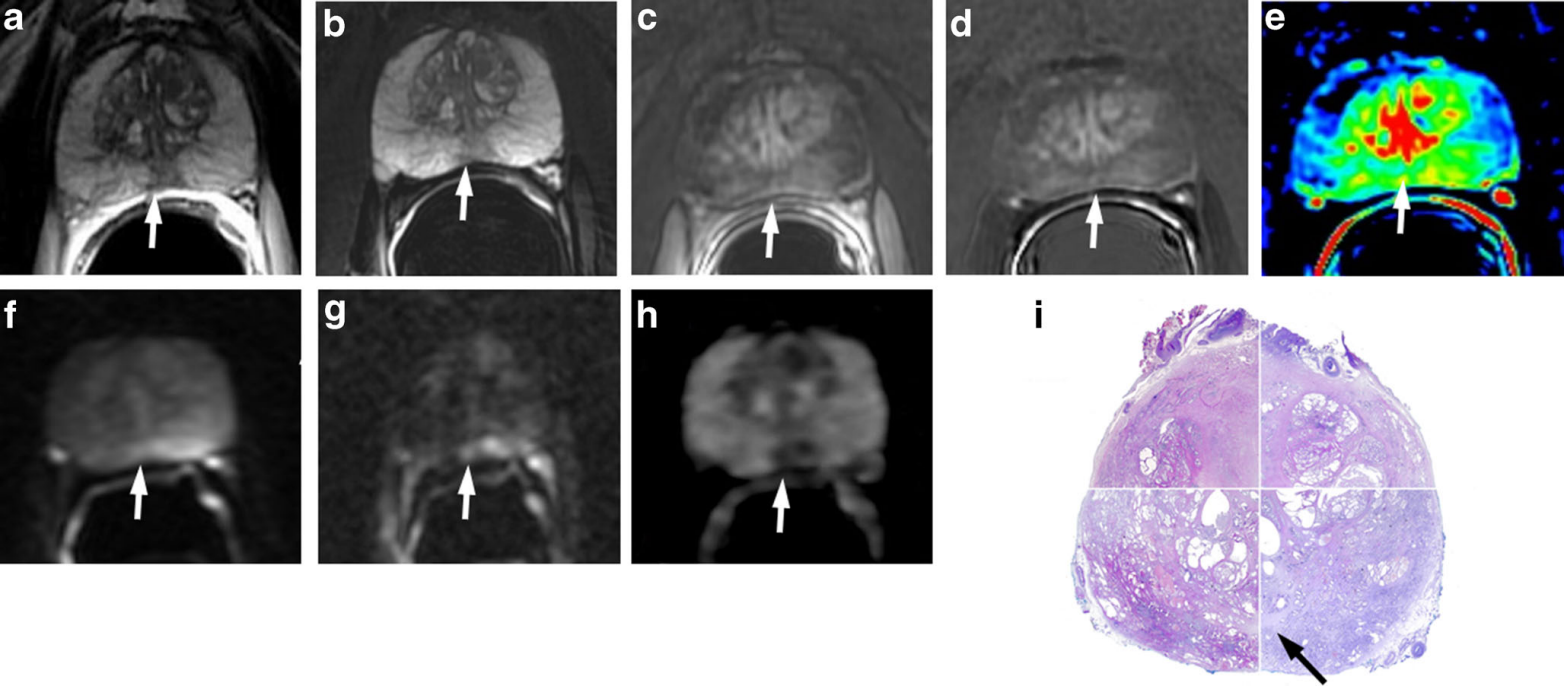
**a** Axial T2-weighted fast spin-echo image (5525/110) and **b** axial T2-weighted fat saturated fast spin-echo image (4941/116) showing a hypointense zone at the mid-peripheral gland in the median posterior location. **c** Axial perfusion, gradient-echo T1-weighted, **d** gradient-echo T1-weighted subtracted image and **e** colour DCE MR map displaying mild enhancement of the hypointense zone. **f** Axial DWI image with b value = 1000 and **g** 3000 mm^2^/sec and h ADC map showing restricted diffusion phenomena of water molecules in the hypointense focus detected on T2-weighted images (*white arrows*). According to the PIRADSv2 scoring system, the score for T2, DCE and DWI is, respectively: 3, + and 4; the overall score is 4, which means highly clinically significant cancer is likely to be present. **i** Photomicrograph shows the area in the posterior middle third of the gland (*black arrows*)

**Fig. 3:**
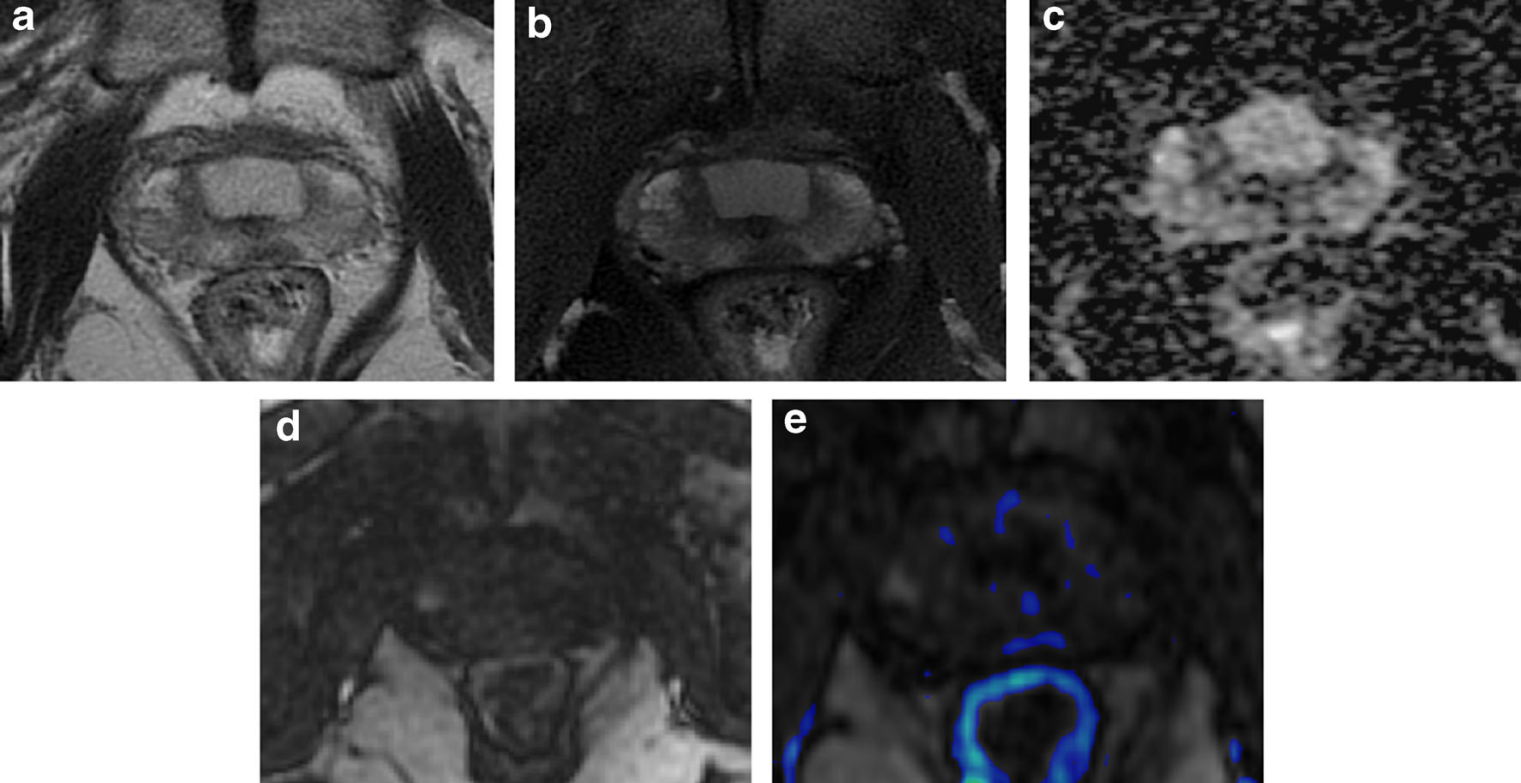
Median posterior hypointense area at the middle third of the gland with no ERC. **a** Axial T2-weighted fast spin-echo image and **b** axial Fat Saturated T2-weighted showing an ill-defined hypointense median area at the middle third of the gland. **c** ADC map showing mild restriction of water molecules diffusivity in the hypointense focus detected on T2-weighted images. **d** Axial perfusion gradient-echo T1-weighted, and **e** colour DCE MR map showing mild enhancement of the hypointense zone

T2-weighted morphologic images can show a median low signal intensity area at the middle third of the posterior zone. This posterior nodular-shaped area may show low ADC values, with less or no contrast enhancement on perfusion sequences. This aspect may be a variant of the previously described “moustache sign”, in which the central zone appears compressed between the transition and peripheral zone, taking a particular kind of shape called a reversed teardrop. The use of the coronal plane is very important to demonstrate the continuity and symmetry of this area with the rest of the central portion (Figs. 2 and 3).

### Transition Zone Prostate Cancer Versus BPH Foci of Stromal Hyperplasia

**Fig. 4:**
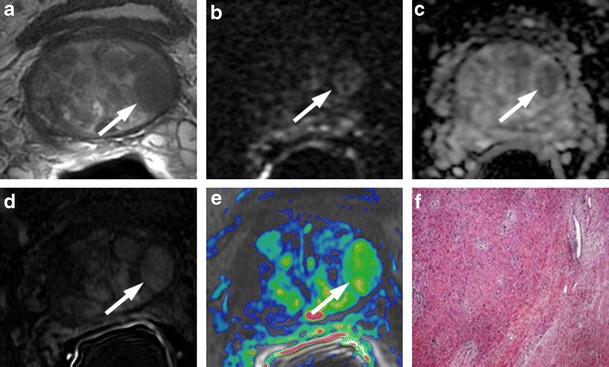
**a** Axial T2-weighted fast spin-echo image (5525/110). **b** Axial DWI image with b value = 1000. **c** ADC map showing mild restricted diffusion phenomena of water molecules in the hypointense focus detected on T2-weighted images (white arrows).**d** gradient-echo T1-weighted subtracted image and **e** colour DCE MR map displaying mild enhancement of the stromal nodule. According to the PIRADSv2 scoring system, the score for T2, DCE and DWI is, respectively: 2, − and 3; the overall score is 3, which means the presence of clinically significant cancer is equivocal. **f** Photomicrograph (50×) showing the BPH stromal nodule

A markedly low signal intensity focus in the transition zone on T2-weighted imaging is usually related to a focus of stromal hyperplasia (SH), but TZ cancers can also show a markedly homogeneous low signal intensity (erased charcoal sign) with ill-defined or well-defined margins and an amorphous, lenticular (lens-like), round or oval shape [23] (Fig. 4). TZ cancers occur with a relatively low frequency, accounting for approximately 30 % of all PCa. They generally have a lower histological grade and remain confined to the prostate, sometimes resulting in a large tumour volume upon detection. The detection rate of TZ cancer on the basis of T2-weighted images alone range between 56 % and 63 % [24–26]. Recent studies reported that quantitative ADC values derived from DWI could improve the diagnostic accuracy in differentiating TZ cancers (0.94–1.37 × 10–3 sec/mm^2^) from BPH nodules of SH (1.34–1.79 × 10–3 sec/mm^2^) [23, 27–30]. The lower the ADC values, the higher the probability of PCa. However, considerable overlap still exists and cannot be used in clinical practice. SH enhances in a way that is similar to TZ cancer; for this reason, we believe that dynamic contrast-enhanced MR imaging parameters alone are not helpful for differentiating TZ cancer from SH. Nevertheless, although quantitative perfusion parameters alone are not effective in the differentiation of TZ cancer from SH, combining the K trans parameter with the ADC shows the possibility of improving this differentiation compared to ADC mapping alone [23]. T2-weighted imaging is actually considered the more reliable sequence in differentiating SH from TZ cancers, by the identification of distinctive SH typical features like the surrounding subtle pseudocapsule, heterogeneous signal intensity, round shape and well-defined margins [16, 31, 32].

### Ectopic BPH Nodule

**Fig. 5:**
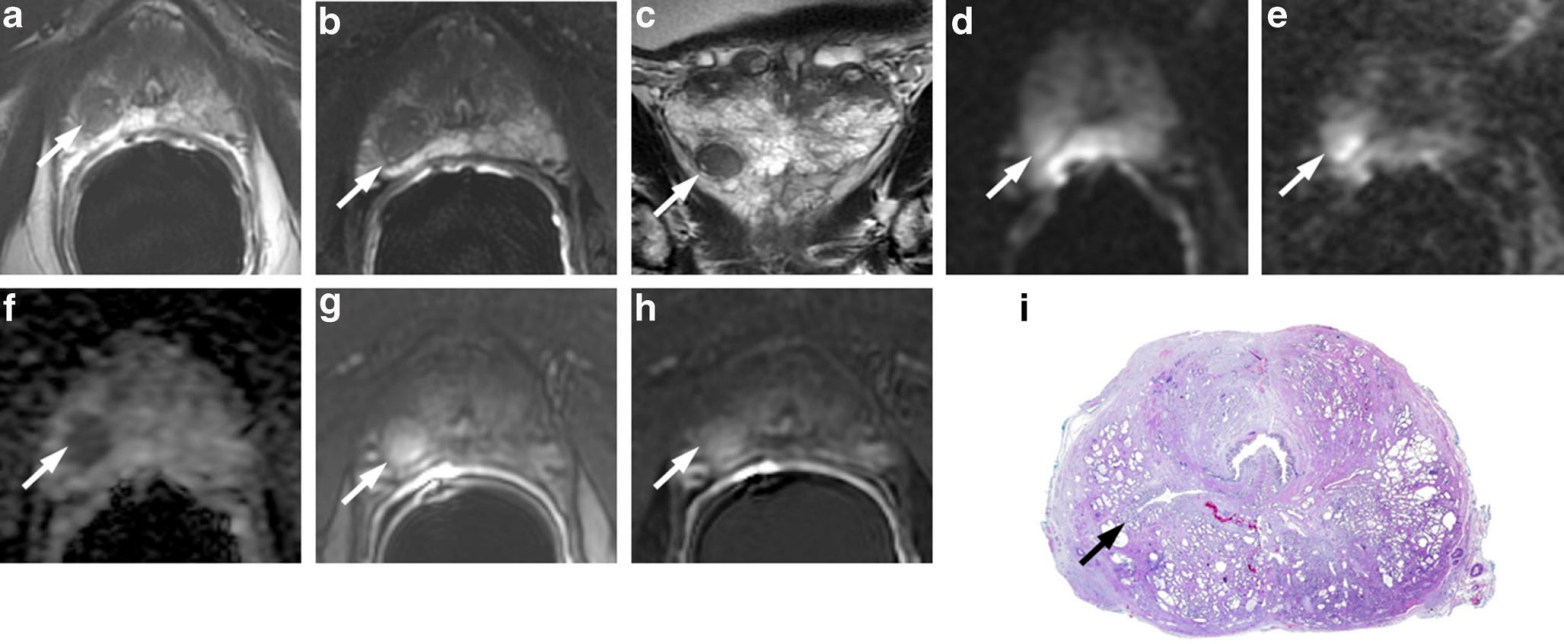
Ectopic stromal BPH nodule in the right peripheral zone (*white arrows*). **a** Axial T2-weighted fast spin-echo image (5045/110), **b** axial T2-weighted fat saturated fast spin-echo image (45461/116) and **c** coronal T2-weighted fast spin-echo image (5565/120) showing a round sharply defined nodule of low signal intensity surrounded by a pseudocapsule in the peripheral zone of the prostate at the right peri-apical location. **d** Axial DWI image with b value = 1000 mm^2^/sec and e = 3000 mm^2^/sec, and **f** ADC map showing remarkable diffusion restricted phenomena. **g** Axial perfusion gradient-echo T1-weighted and h gradient-echo T1-weighted subtracted image showing avid enhancement of the nodule. According to the PIRADSv2 scoring system, the score for T2, DCE and DWI is, respectively: 4, + and 3; the overall score is 3, which means the presence of clinically significant cancer is equivocal. **i** Pathologic correlation after prostatectomy yielded BPH (*black arrows*)

**Fig. 6:**
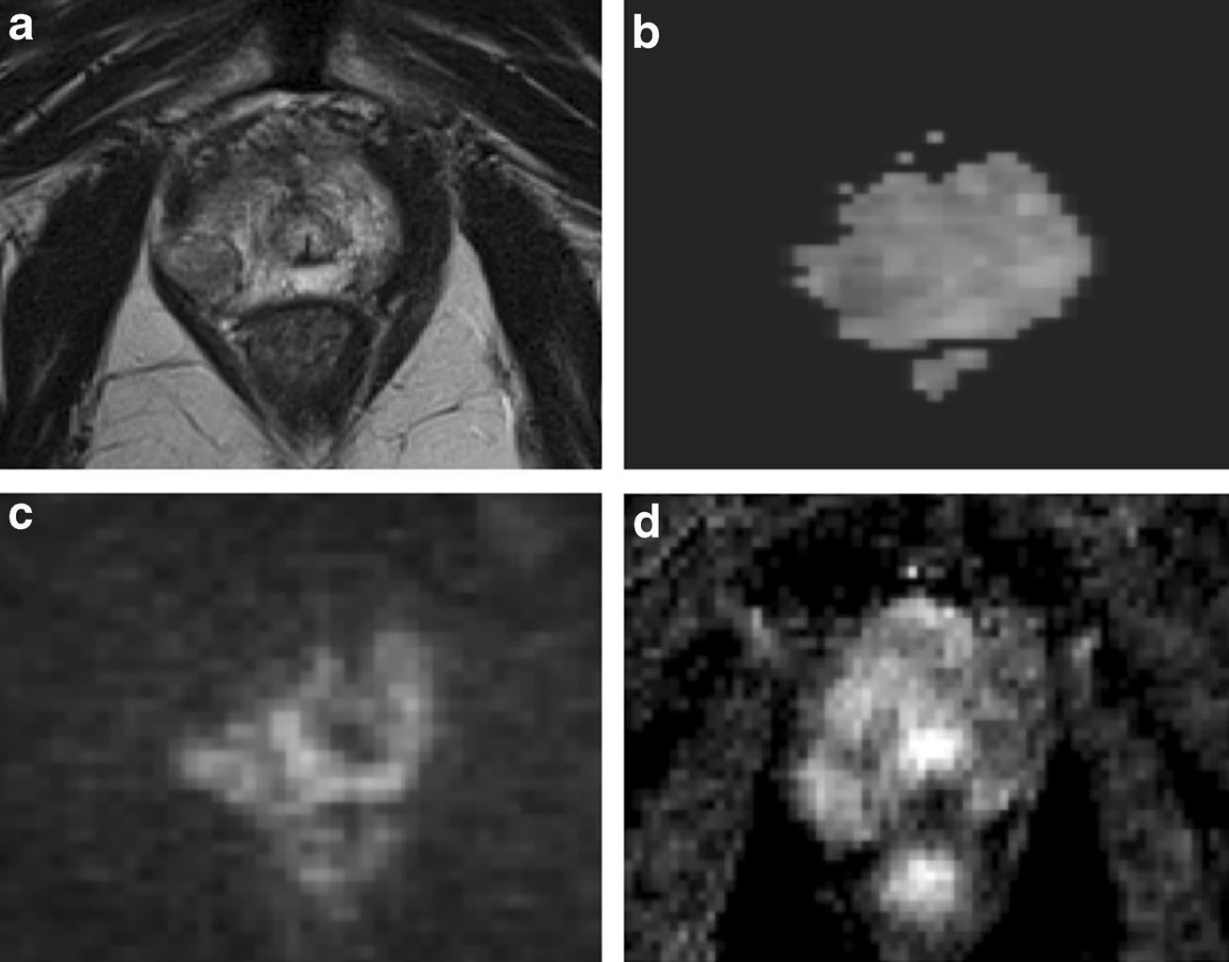
Ectopic nodule in the right peripheral zone with no ERC. **a** Axial T2-weighted fast spin-echo image showing an hypointense well circumscribed nodule in right peripheral zone with an evident peripheral capsule. **b** ADC map and **c** axial DWI image with b value = 3000 mm^2^/sec, showing no significant restriction of water diffusivity. **d** Axial perfusion gradient-echo T1-weighted image shows marked and early enhancement simultaneous to other nodule constituents of the adenoma

BPH is a nodular, regional growth with a variegated gross appearance resulting from the inhomogeneous and irregular mixture of glandular and stromal tissue. Although BPH nodules are mostly located centrally in the enlarged TZ, they sometimes may arise in the PZ of the prostate. A focal peripheral nodule of remarkably low, intermediate or sometimes high signal intensity on T2-weighted images characterized by sharply-defined margins, round or oval shape and surrounded by a subtle pseudocapsule should not be mistaken for a peripheral PCa. T2-weighted morphologic images can sometimes show a median low signal intensity area at the middle third of the posterior zone, continuous to the posterior portion of an adenoma. This posterior bulging portion of BPH can show a nodular shape, low ADC values, and an enhancement similar to the central portions of the adenoma. The use of sagittal and/or coronal planes is very important to demonstrate the continuity with the adenoma. Ectopic BPH nodules enhance in a way that is similar to PCa; for this reason, DCE-MRI is not helpful for distinguishing between ectopic BPH nodules and peripheral foci of PCa. Quantitative ADC values could improve the diagnostic accuracy in discerning peripheral PCa from ectopic BPH nodule: the higher the ADC values, the higher the likelihood of ectopic BPH nodule (Figs. 5 and 6) Ectopic BPH nodules sometimes contain tiny bright spots corresponding to dilated acini, and this is a characteristic that can be used to distinguish them from PZ PCa.

### Granulomatous Prostatitis after Intravesical Instillation of BCG

**Fig. 7:**
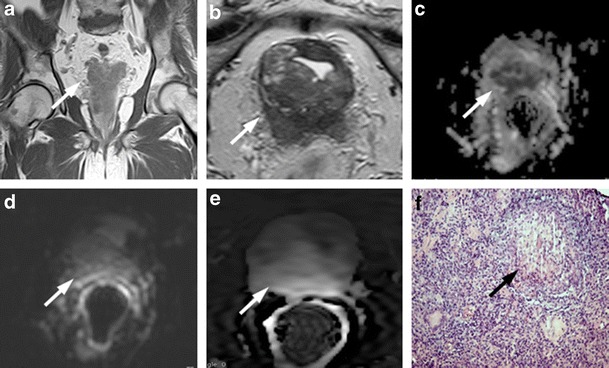
**a** Coronal T2-weighted fast spin-echo image (5525/110) and **b** Axial T2-weighted fast spin-echo image (5525/110) showing diffuse peripheral hypointensity in a patient who underwent intravesical instillation of BCG (*white arrows*). **c** ADC map showing high restricted diffusion phenomena of water molecules in the hypointense posterior area detected on T2-weighted images. **d** Axial DWI image with b value = 1000 and **e** gradient-echo T1-weighted subtracted image show slight enhancement of the area. **f** Histological picture shows diffuse granulomatous inflammation with multinucleated giant cells adjacent to a prostatic gland, (100×, H&E) (*black arrow*)

The granulomatous reaction that occurs in prostate tissue following the topical instillations of BCG after resection of bladder cancer can present MRI features very similar to those found in prostate cancer [33]. Granulomatous prostatitis, in the most common pattern, presents in T2-weighted images as an area of low SI, with ill-defined margins and variable shape, from nodular to band-like. ADC maps in the area of granulomatous prostatitis may present restriction of diffusion with even very low ADC values, due to the high cellular density. DCE-MRI presents with moderate enhancement, not intense, as it generally occurs in prostate cancers (Fig. 7).

### Hypertrophic Anterior Fibromuscular Stroma

**Fig. 8:**
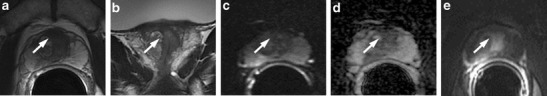
Hypertrophic anterior fibromuscular stroma (*white arrows*). **a** Axial T2-weighted fast spin-echo image (5045/110), **b** coronal T2-weighted fast spin-echo image (5565/120) showing hypertrophic area of low signal intensity between the small transition zone lobes. **c** Axial DWI image with b value = 1000 mm^2^/sec and **d** ADC map showing no diffusion restricted phenomena. **e** Axial perfusion gradient-echo T1-weighted subtracted image showing late mild enhancement of the zone. According to the PIRADSv2 scoring system the score for T2, DCE and DWI is, respectively: 4/5, − and 2; the overall score is 4 (based on dominant sequence), which means the presence of clinically significant cancer is likely to be present

Anterior fibromuscular stroma (AFMS), according to the McNeal zonal anatomy distinction, is the most anterior portion of the gland, situated between the two lobes that constitute the transition zone (TZ), which often appears to be flattened by them. That zone is basically formed by muscle cells intermingled with dense connective tissue, without the presence of glandular tissue. Because of its histological constitution on T2-weighted images, it appears homogeneously hypointense, with a signal intensity similar to that of PCa. It is possible to find in some cases, especially in the presence of a modestly sized adenoma, a sometimes asymmetrical hypertrophy of the AFMS, which can mimic the presence of a PCa. In this case, DWI and DCE-MRI become essential to distinguish a zone of hypertrophic AFMS, because such a zone does not show restriction on DWI or enhancement on DCE-MRI [34] (Fig. 8).

### Periprostatic Venous Plexus and Neurovascular Bundle

**Fig. 9:**
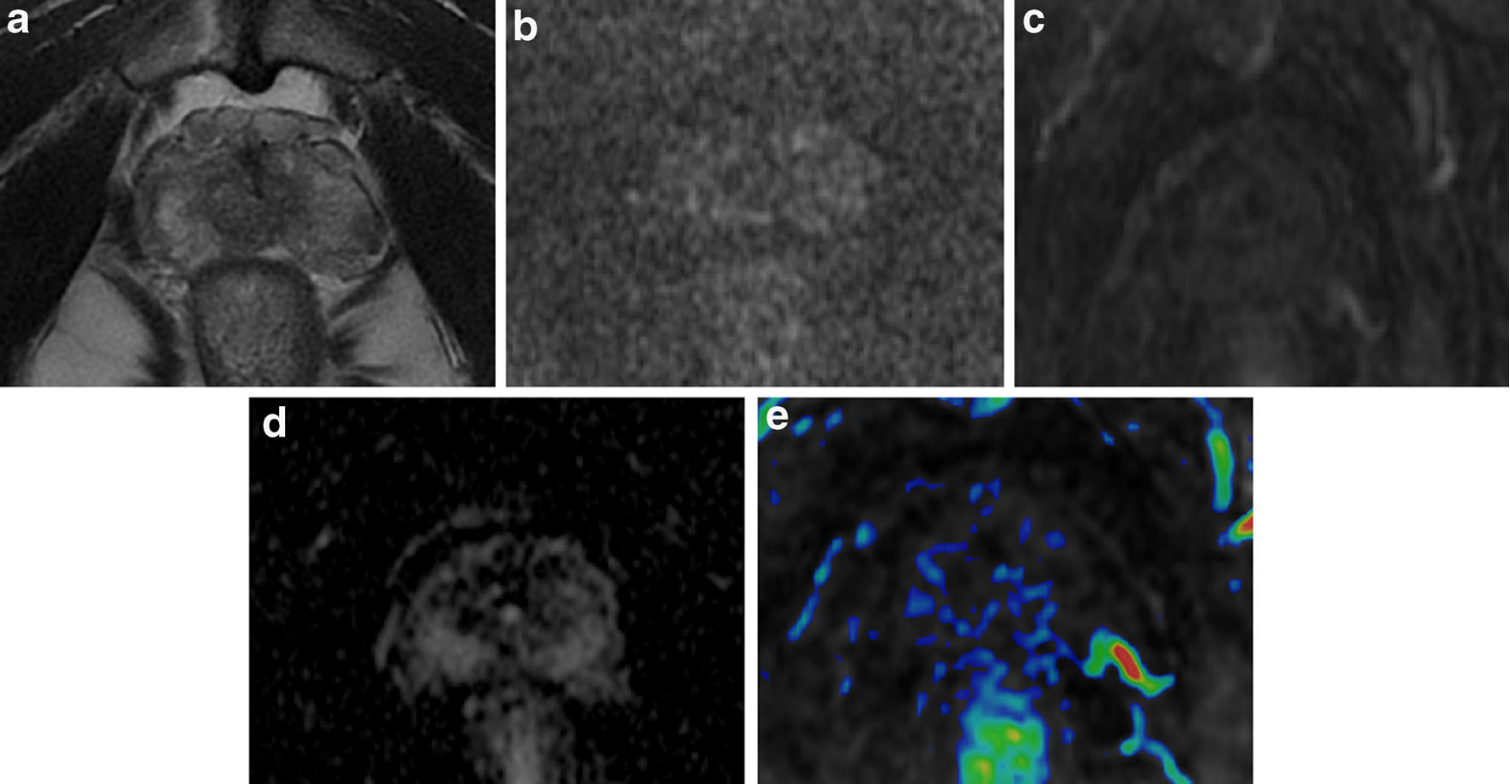
Prominent peri-prostatic bundle with no ERC. **a** Axial T2-weighted fast spin-echo image showing an ill-defined hypointense area in posterior left peripheral zone, suspected for cancer. **b** Axial DWI image with b value = 3000 mm^2^/sec, and **c** ADC map image showing no areas of significant restriction of water diffusivity. **d** Axial perfusion gradient-echo T1-weighted image, and **e** colour DCE MR map showing no enhancing areas in left peripheral zone with and enhancing neuro-vascular bundles adherent to the glandular profile. According to he PIRADSv2 scoring system, the score for T2, DCE and DWI is, respectively: 2, + and 4; the overall score is 4 (based on dominant sequence), which means that the presence of clinically significant cancer is equivocal

The proximity of both the periprostatic venous plexus and neurovascular bundle (NVB) to the peripheral zone may create a clinical challenge in assessing for focal peripheral zone lesions. The periprostatic venous plexus or NVB will have a discrete rounded appearance when viewed en face on axial slices. In addition, these structures exhibit decreased T2 signal intensity and a signal void on the ADC map, and could be erroneously considered suspicious for a lesion within the peripheral zone. Nevertheless, the periprostatic venous plexus or NVB may be correctly identified given the typical location coursing along the lateral margin of the peripheral zone and rounded contour if viewed en face on an individual axial slice, combined with the tubular morphology when tracked across consecutive slices and potential visualization of a similar-appearing structure on the contralateral side. Generally, the T2-weighted images, given their higher in-plane spatial resolution, are helpful to show that a potential lesion identified on DWI or DCE-MRI represents the normal periprostatic venous plexus or NVB. In addition, the expected delayed venous enhancement of the periprostatic venous plexus (and possibly of the NVB, given its intimate association with small venous structures) may be more readily apparent on inspection of the pre-processed dynamic contrast-enhanced images than on the post-processed parametric perfusion maps, given the greater anatomic clarity of the raw contrast-enhanced images [21] (Fig. 9).

## Pitfalls in the Staging

### Local Staging

At the apex: Pseudo T3a stage is caused by an anatomical variation of the shape of the apex, when PZ prostatic tissue is present along the posterior aspect of the distal urethra. When involved by tumour, it can simulate subapical T3 disease.

At the base: bulging BPH nodules along the root of seminal vesicles simulates T3b stage.

Any location: granulomatous prostatitis commonly infiltrates the periprostatic fat and simulates T3a disease.

At the postero-lateral portion of the gland: periprostatic venous plexus and neurovascular bundle.

The pitfalls mentioned above are representative of T3 vs. T2, and overlap with pitfalls in primary diagnosis.

### Benign Bone Findings Mimicking Prostate Cancer Metastases

**Fig. 10:**
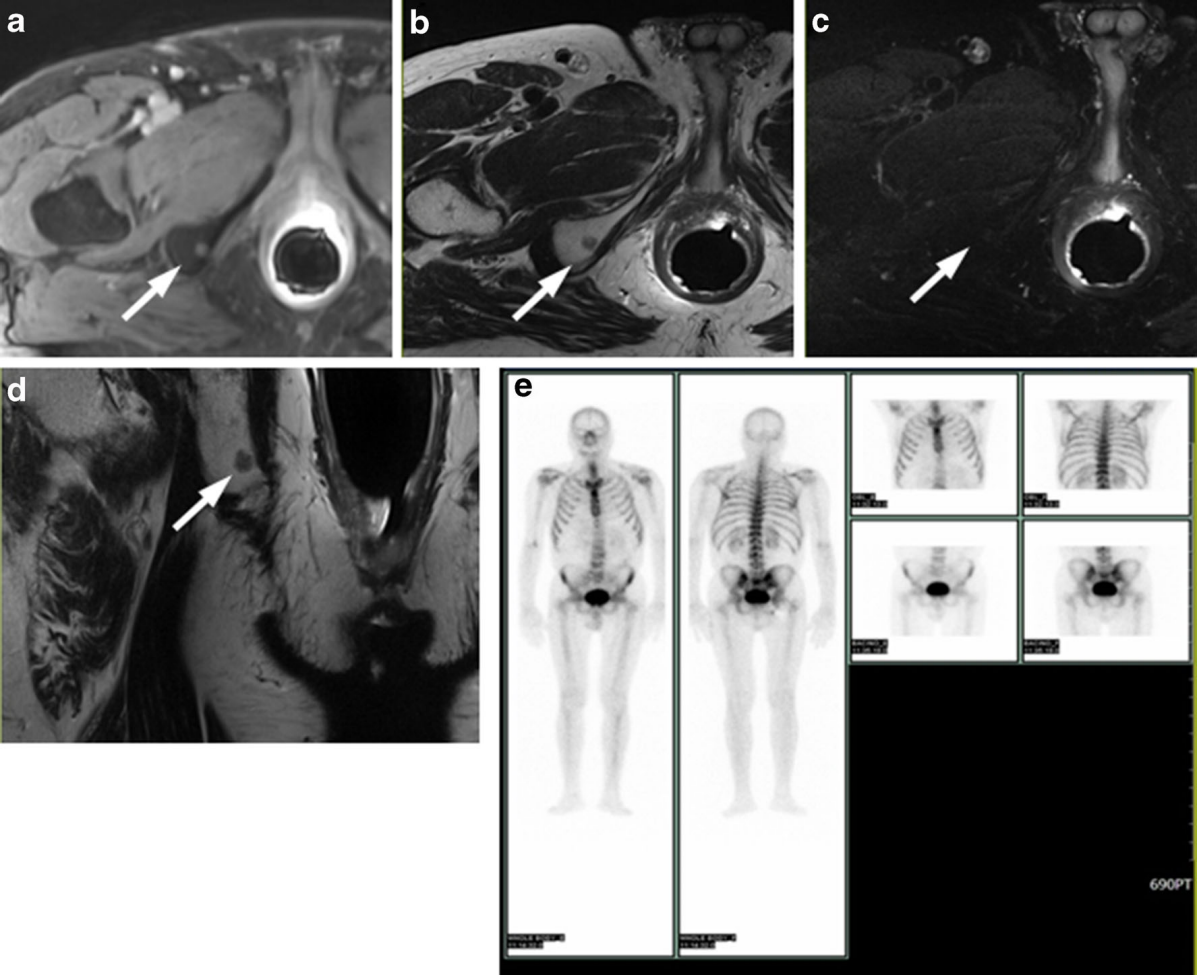
**a** Axial post-contrast T1-weighted fat saturated 3D FLASH (VIBE) axial image showing a small enhancing area in the right ischiopubic ramus. **b** Axial T2-weighted fast spin-echo (6330/115) and **d** coronal T2-weighted (5230/114) images display a small round hypointense area, which appears slightly hyperintense (*white arrows*) on **c** axial T2-weighted fat saturated image (8450/115). **e** Bone scan showing uptake of radiotracer in the right ischiopubic ramus. All these findings are consistent with bone metastasis. In addition, the reference standard was the drop of PSA value after hormonotherapy (from 18 ng/mL to 4.5 ng/mL)

**Fig. 11:**
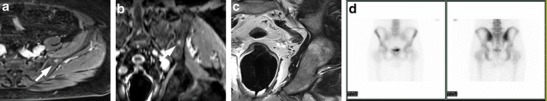
**a** Post-contrast T1-weighted fat saturated 3D FLASH (VIBE) axial image and **b** coronal T1-VIBE reconstructed image showing a small enhancing focus in the left iliac bone. **c** No bone alterations detected in the corresponding site on coronal T2-weighted fast spin-echo image (7940/112) (*white arrows*). **d** No areas with uptake of radiotracer are displayed at bone scan. These findings exclude the hypothesis of bone metastasis

**Fig. 12:**
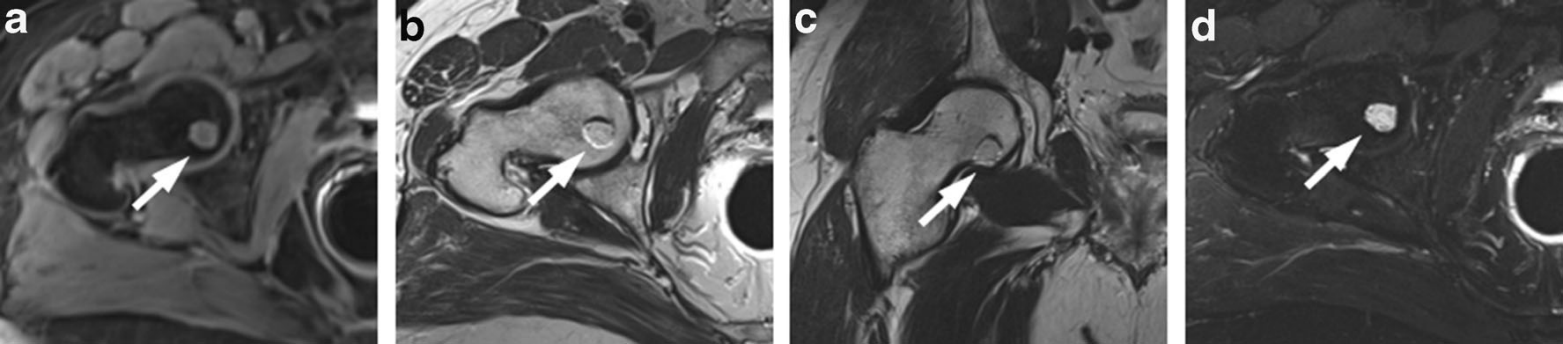
**a** Axial post-contrast T1-weighted fat saturated 3D FLASH (VIBE) axial image showing a round, well-defined hypervascular lesion in the right femoral head (*white arrows*). **b** Axial T2-weighted fast spin-echo (6200/112), **c** coronal T2-weighted (4110/112) and **d** axial T2-weighted, fat saturated spin-echo (8300/112) images display a round, lobulated, markedly hyperintense lesion surrounded by a sclerotic rim. These findings are consistent with enchondroma

Focal enhancing zones in the pelvic bone structures detected on contrast-enhanced T1-weighted images are usually suspicious for prostate cancer metastases (Fig. 10). A careful analysis of morphology of such findings on T2-weighted images and of behaviour in diffusion images is mandatory to exclude malignancy. For example, these focal enhancing zones may represent hyperplastic islands of bone-marrow (which appear as an isointense or slight hypointense well or ill-defined area on T2-weighted images) (Fig. 11), or benign bone tumours like enchondroma (which appears as a lobulated lesion with polycyclic margins, internal septations, high signal intensity on T2-weighted images with small foci of low signal intensity due to calcified chondroid matrix (Fig. 12).

### Controversial on Lymph Nodes

Another role of MRI in the evaluation of patients with PCa is the detection of pathologically involved pelvic lymph nodes. According to standard criteria on conventional MRI, lymph nodes are classified as malignant if the short-axis diameter is elongated and exceeds 10 mm, or is rounded and exceeds 8 mm. Unfortunately, it could happen that these kind of lymph nodes are disease free, or that lymph nodes less than 10 mm considered as non metastatic are pathologically involved at histology [35]. The use of lymphotropic, superparamagnetic iron oxide nanoparticle contrast agents can improve the Sensitivity (Se) and Specificity (Spe) of MRI in the detection of lymph node metastases. These nanoparticles have a monocrystalline, inverse spinel, superparamagnetic iron oxide core, contain a dense packing of dextrans to prolong their time in circulation, and are avidly internalized by macrophages within lymph nodes and result in changes in the magnetic properties detectable by MRI [36] After injection of nanoparticles, nodes could be considered malignant when one of the following three criteria is present: a decrease in signal intensity (SI) of less than 30 % on T2W or gradient-echo sequences; a heterogeneous SI (giving the entire node a mottled appearance), discrete focal defects (isolated islands of high SI), or both; a central area of hyperintensity (excluding a fatty hilum), but a peripheral decrease in SI. In a study of 80 patients with T1-3 PCa who subsequently underwent surgical lymph node resection or targeted lymph node biopsy for a total of 334 lymph nodes evaluated with direct MRI-histological correlations, conventional MRI detected pathologically involved nodes with a Se of 35.4 % and Spe of 90.4 %, compared with 90.5 % Se and 97.8 % Se for MRI with nanoparticle contrast agents [36].

## Pitfalls after Treatment

Radical prostatectomy has been performed for more than a century [37], and remains the most common treatment choice in patients with organ-confined prostate cancer [38]. It involves the removal of the entire prostate, the seminal vesicles, and the ampullary portions of the vasa deferentia, with the formation of a vesicourethral anastomosis. Whenever possible, the surgical procedure is tailored to preserve the neurovascular bundles responsible for erectile function as well as the external sphincter responsible for urinary continence. The likelihood of simultaneous achievement of all three desired outcomes (cancer-free status, continence, and potency, sometimes called the “trifecta”) after radical prostatectomy has been reported to be 60 %–91 % after 18–24 months [39–41].

### Residual Glandular Tissue, Fibrosis and Granulation Tissue

**Fig. 13:**
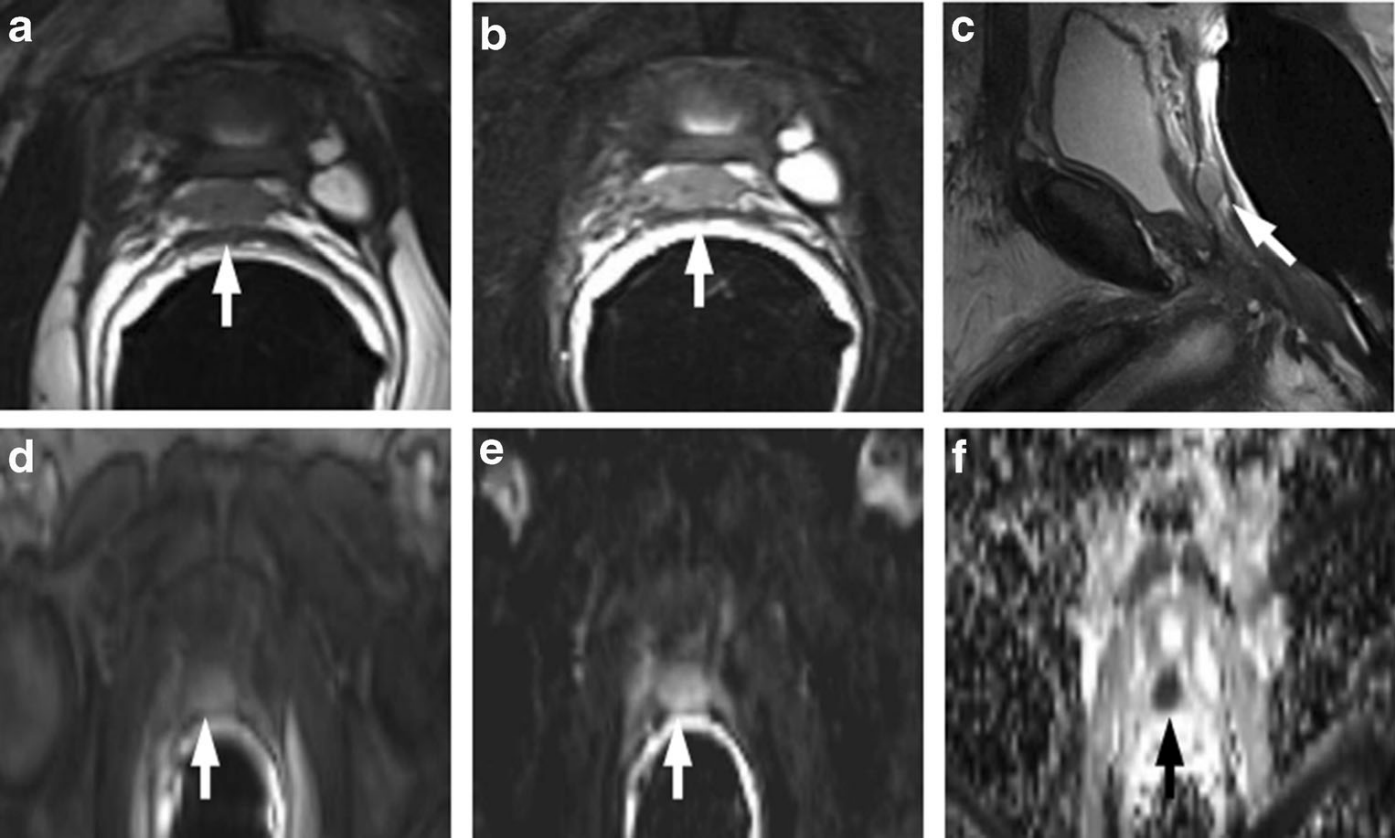
MR images of a 64-year-old man with prostate-specific antigen progression (PSA serum level 0.75 ng/mL) after radical retropubic prostatectomy, with suspected local recurrence. **a** Axial T2-weighted fast spin-echo (6200/112), **b** axial T2-weighted fat saturated fast spin-echo (8300/112) and **c** sagittal T2-weighted fast spin-echo (4640/112) show a solid tissue on posterior perianastomotic location in front of the rectal wall at about 40 mm from the ureteral meatus, which is slightly hyperintense compared to pelvic muscles (*white arrows*). **d** Axial Gradient-echo T1-weighted image and **e** Gradient-echo T1-weighted subtracted image showing a remarkable enhancement of the pathological tissue **f** Axial ADC map reconstructed from images obtained at b values of 0, 500 and 1000 s/mm show a dark area corresponding to the abnormal hyperintense tissue seen on T2-weighted images (*black arrow*). All these findings are consistent with local recurrence

**Fig. 14:**
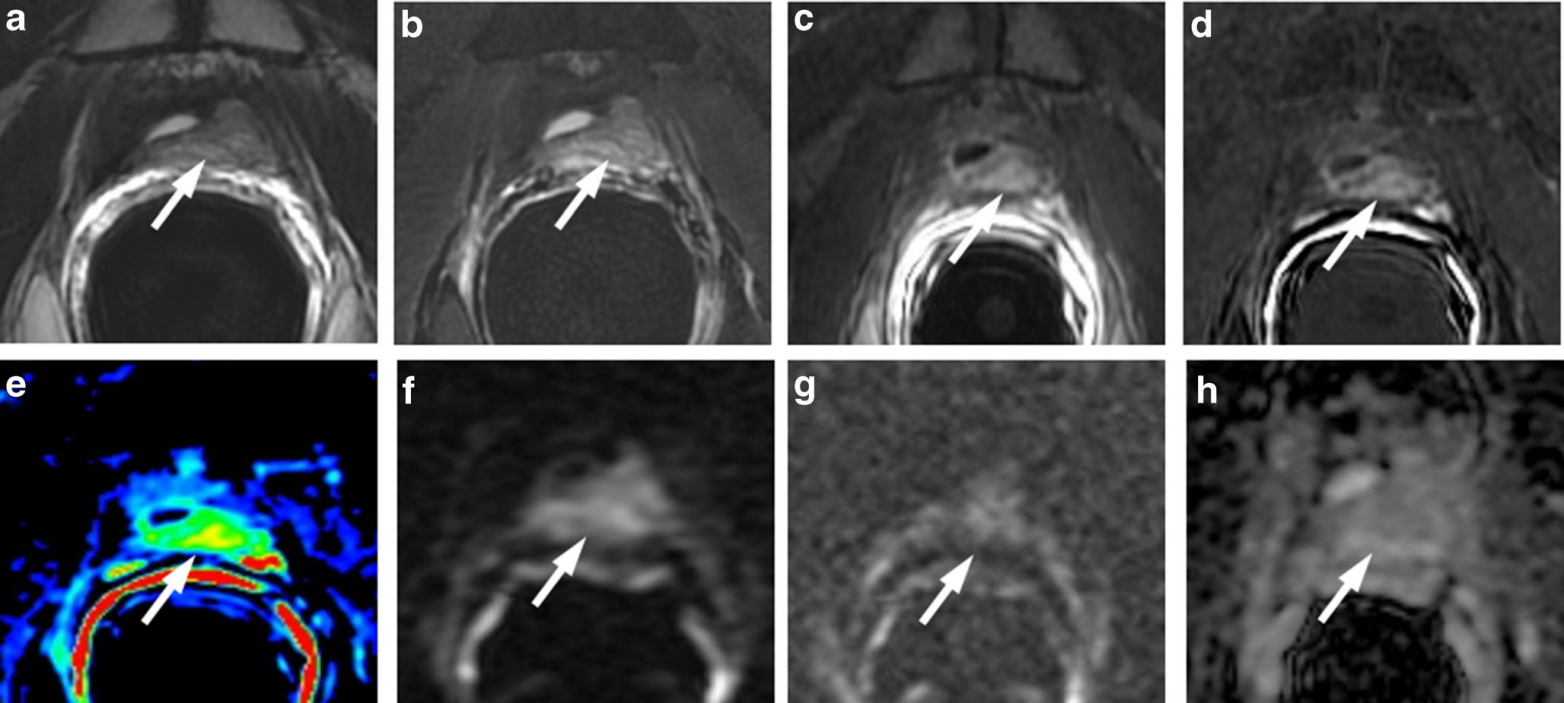
MR images of a 69-year-old man with prostate-specific antigen progression (PSA serum level 1.06 ng/mL) after radical retropubic prostatectomy, with suspected local recurrence. **a** Axial T2-weighted fast spin-echo (5525/109) and **b** axial T2-weighted fat saturated fast spin-echo image (5981/116) show a solid tissue on posterior perianastomotic location in front of the rectal wall at about 33 mm from the ureteral meatus which is hyperintense compared to pelvic muscles (*white arrows*). **c** Axial gradient-echo T1-weighted, **d** gradient-echo T1-weighted subtracted image and **e** DCE MR colour map showing a positive contrast enhancement of the abnormal tissue. **f** Diffusion weighted images with a b value of 1.000,**g** 3.000 s/mm^2^ and **h** ADC map show no diffusion restricted phenomena, consistent with residual glandular tissue

The presence of soft tissue in the prostatectomy bed that is isointense to muscle on T1-weighted images and slightly hyperintense to muscle on T2-weighted images should be considered as strongly suggestive of local recurrence. The most common site of postoperative local recurrence is the vesicourethral anastomosis [42]. Other common sites of local recurrence are retrovesical (between urinary bladder and rectum), within retained seminal vesicles, or at the anterior or lateral surgical margins of the prostatectomy bed (e.g., abutting the levator ani muscles) [43]. In most cases, local recurrence can be readily distinguished from normal postoperative fibrosis, which is of low signal intensity compared with muscles on images obtained with all sequences (Fig. 13). However, if the MRI was performed shortly after surgery, there is a possibility of finding the granulation tissue within the surgical site, particularly in the perianastomotic region where it can mimic the appearance of tumour recurrence [43]. In these circumstances, both T2-weighted and contrast-enhanced MR imaging have a limited role. Recurrent tumour tends to be slightly hyperintense on T2-weighted sequences and avidly enhances in the arterial phase with wash-out during the venous phase, while post-operative granulation-tissue may present the same features found on T2 and DCE [14–16]. Sometimes it is very difficult to distinguish between a local recurrence nodule and residual glandular healthy tissue, because both appear slightly hyperintense compared to muscles on T2-weighted images and may be hypervascular on perfusion images. In these cases, the differential diagnosis may be achieved by means of MRSI and DWI (Fig. 14). The ratio of choline (Cho) plus creatine (Cr) to citrate (Ci) in the voxels of interest may help in disclosing the most likely origin of the tissue in the post-prostatectomy bed. Fibrotic/scar tissue is likely when Cho + Cr/Ci < 0.2; residual healthy prostatic gland tissue when Cho + Cr/Ci > 0.2 and < 0.5; probably recurrent prostate cancer tissue when Cho + Cr/Ci > 0.5 and < 1; and definitively recurrent prostate cancer tissue when Cho + Cr/Ci > 1 [43]. The ADC values are a very useful tool to distinguish between loco-regional relapse and residual glandular tissue, and also to express a likelihood of the aggressiveness of the local recurrence. In a recent study, Panebianco et al. found that the mean and standard deviation of ADC values were 0.5 ± 0.23 mm^2^/s for high-grade recurrence, 0.8 ± 0.09 mm^2^/s for intermediate-grade recurrence, 1.1 ± 1.17 mm^2^/s for low-grade relapse and higher than 1.3 mm^2^/s (mean ADC values 1.4; range 1.3–1.7) for residual glandular healthy tissue [17].

### Retained Seminal Vesicles

**Fig. 15:**
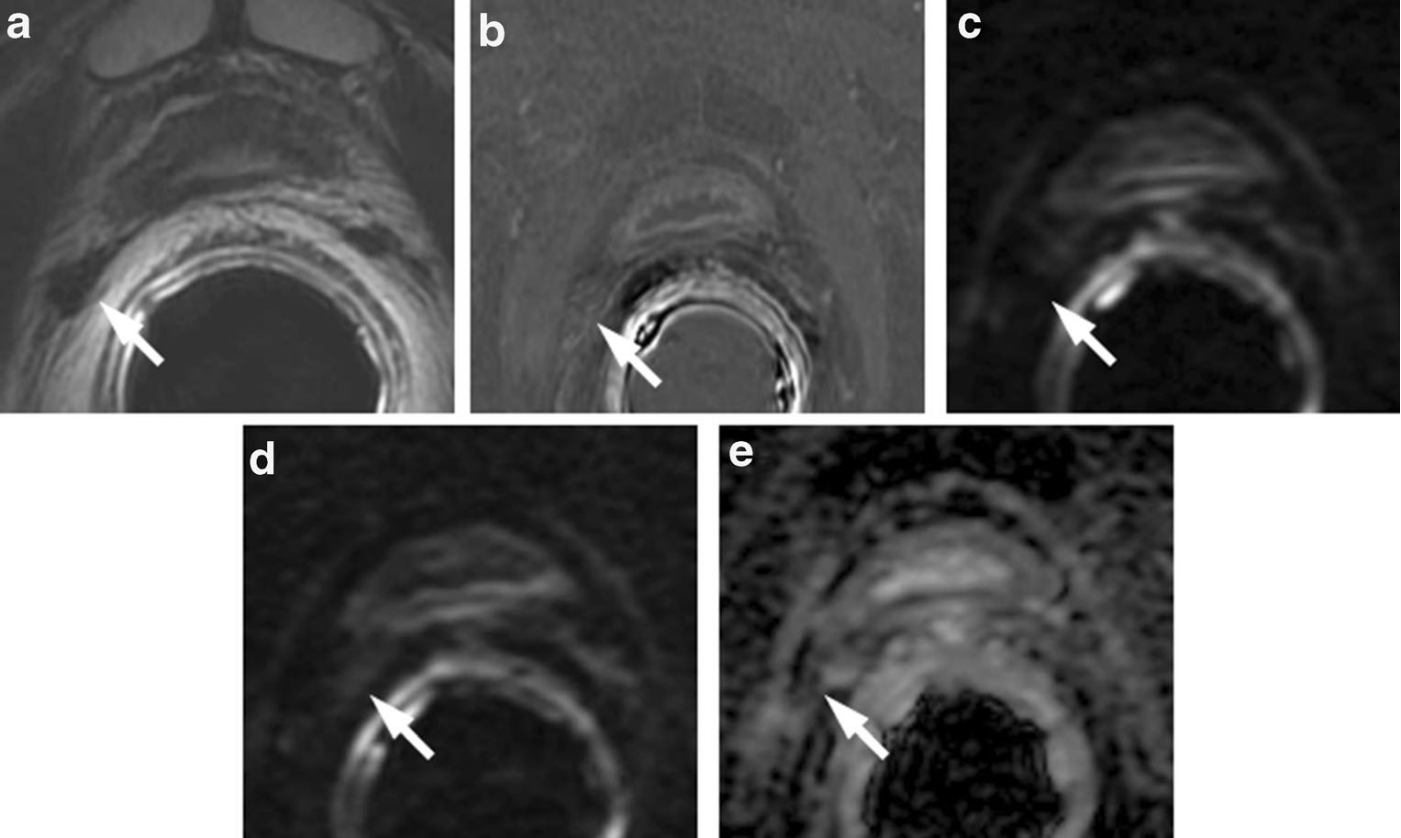
MR images of a 64-year-old man with prostate-specific antigen progression (PSA serum level 0.9 ng/mL) after radical retropubic prostatectomy, with suspected local recurrence. **a** Axial T2-weighted fast spin-echo image shows, on the zone of right seminal vesicle, a nodular tissue (*white arrows*) with signal intensity similar to that of muscle. **b** Axial gradient-echo T1-weighted subtracted image shows no signs of enhancement on the same location as the solid nodular tissue seen on trasverse T2-weighted images. **c** Diffusion weighted images with a b value of 1.000 and **d** 3.000 s/mm^2^ and **e** ADC map show no diffusion restricted phenomena on corresponding site. These findings are consistent with seminal vesicle remnants with fibrotic evolution

**Fig. 16:**
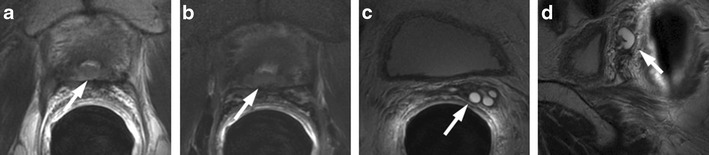
MR images of a 63-year-old man with prostate-specific antigen progression (PSA serum level 1.9 ng/mL) after radical retropubic prostatectomy. **a** Axial T2-weighted fast spin-echo (6006/109) and **b** axial T2-weighted fat saturated fast spin-echo image showing residual glandular tissue. **c** Axial T2-weighted fast spin echo (6006/109) and **d** sagittal T2-weighted fast spin echo (5059/120) showing retained seminal vesicles, which appear as high signal intensity tubular structures (*white arrows*)

Retained seminal vesicles are observed in approximately 20 % of patients after prostatectomy [44]. On computed tomography (CT), retained seminal vesicles may be confused with soft-tissue recurrence; however, the vesicles are usually easily recognizable on MR images, as they tend to maintain their normal convoluted tubular appearance and high intraluminal signal intensity on T2-weighted images [45]. Alternatively, low signal intensity, relative to that of muscle, on both T1 and T2-weighted images, presumably as a result of fibrosis, may be seen in seminal vesicle remnants [44] (Fig. 15). Careful analysis of DCE and DWI images is required to exclude local recurrence at the site previously occupied by the seminal vesicles, because it shows no marked enhancement and no restricted diffusion of water molecules (Fig. 16).

### Sealed Off Veins

**Fig. 17:**
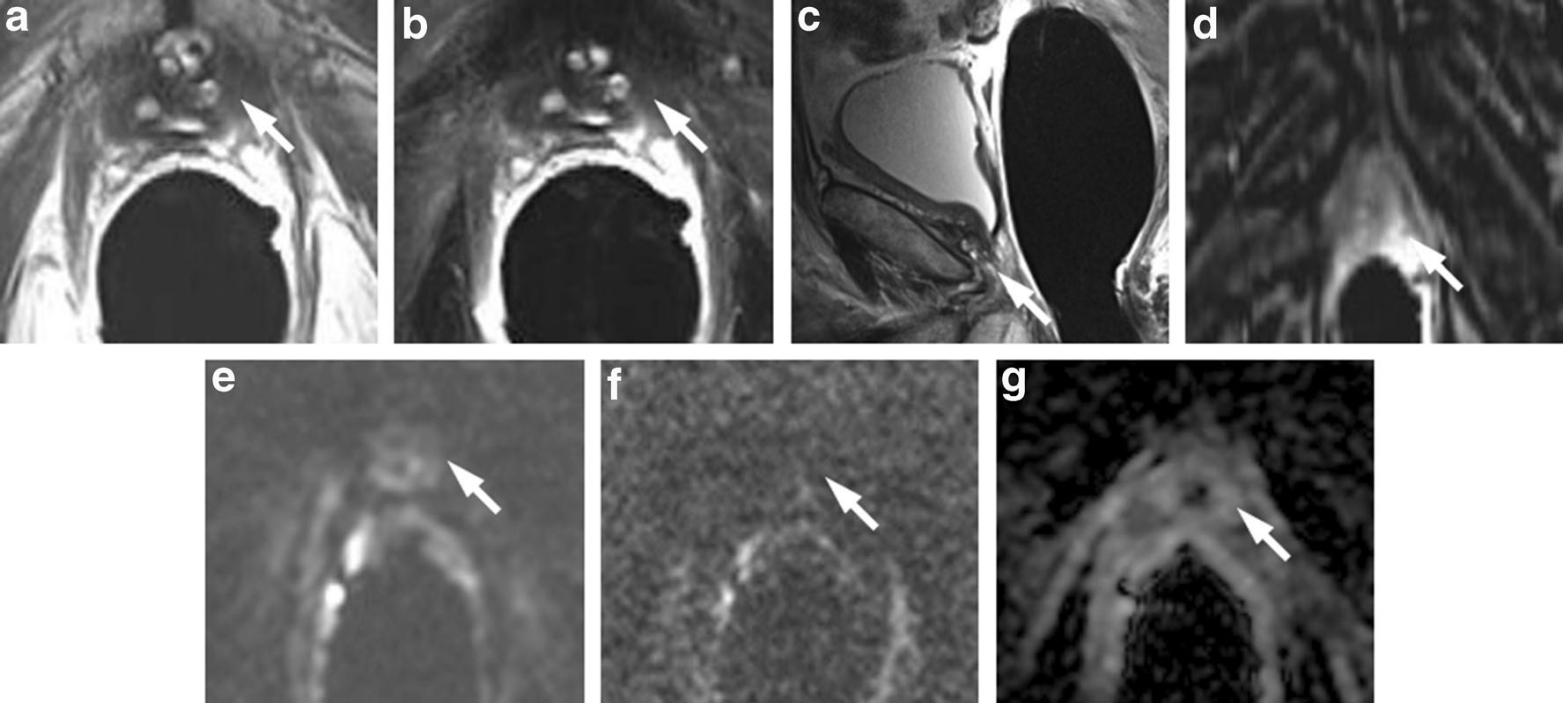
MR images of a 61-year-old man with prostate-specific antigen progression (PSA serum level 0.62 ng/mL) after radical retropubic prostatectomy, with suspected local recurrence. **a** Axial T2-weighted fast spin-echo (5170/112), **b** axial T2-weighted fat saturated fast spin-echo (6920/112) and **c** sagittal T2-weighted fast spin-echo (4730/114) image showing a hyperintense lobulated tissue on anterior perianastomotic location posterior to the pubic symphysis **d** Axial perfusion Gradient-echo T1-weighted subtracted image showing no signs of enhancement of the suspicious pathological tissue. **e** Diffusion-weighted images with a b value of 1.000, *f* 3.000 s/mm^2^ and *g* ADC map show no diffusion restricted phenomena. The hyperintese lobulated tissue detected in T2-weighted images represents a prominent sealed off venous plexus (*white arrows*)

Sometimes, a prominent sealed off venous plexus in the post-prostatectomy fossa adjacent to the vesico-urethral anastomosis appears as a small lobulated tissue mass with high signal intensity on T2-fat saturated images; this tissue does not show enhancement after intravenous injection of contrast media and diffusion restriction of water molecules at DWI (Fig. 17).

### Prominent Periprostatic Venous Plexus

**Fig. 18:**
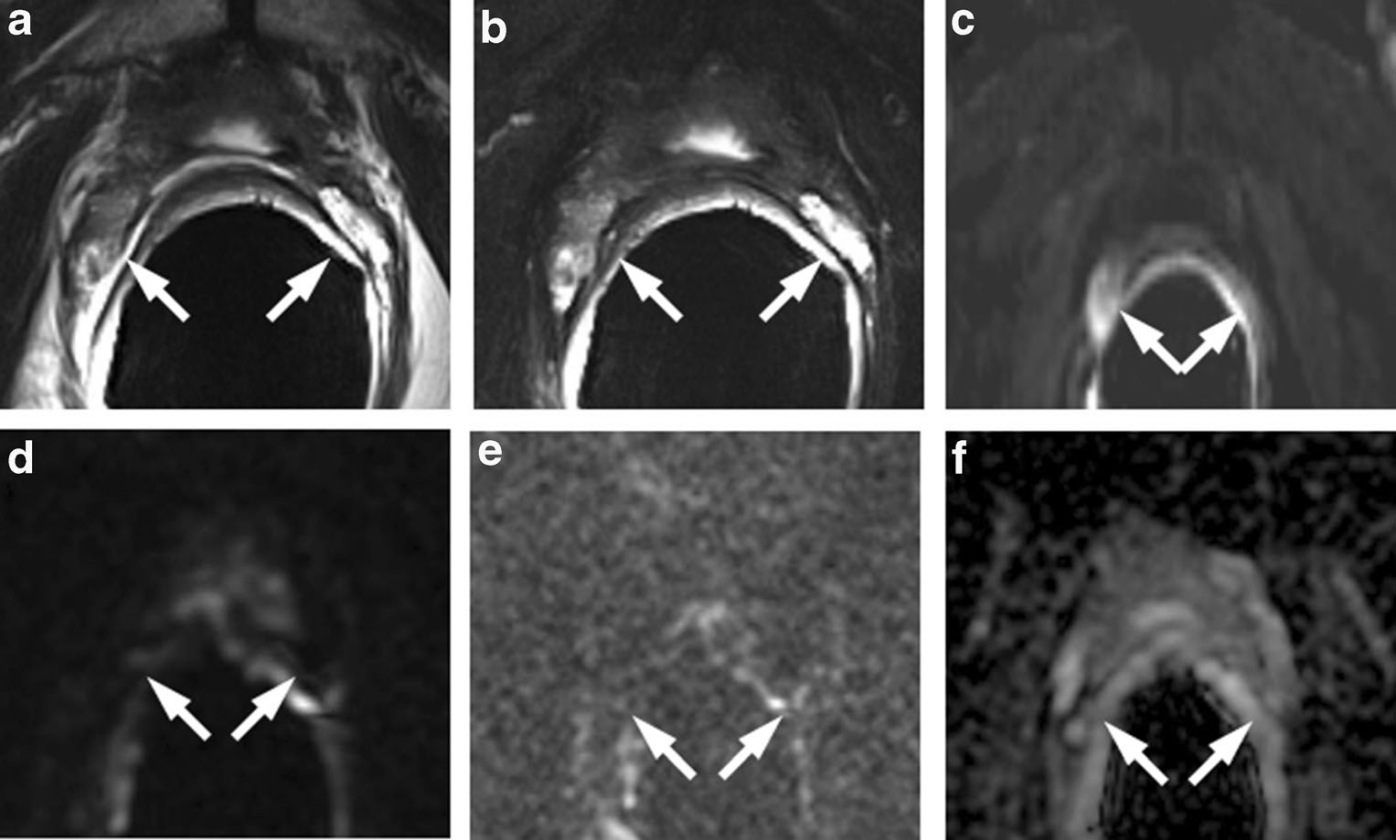
**a** Axial T2-weighted fast spin-echo (6330/115) and **b** axial T2-weighted fat saturated fast spin-echo image (8450/115) show, on the zone previously occupied by the right seminal vesicle, a slightly hyperintense lobulated tissue. **c** Gradient-echo T1-weighted subtracted image shows a well-defined area of marked enhancement (arrows) on the same location as the lobulated tissue seen on T2-weighted images. **d** Diffusion weighted image with a b value of 1.000 and *e* 3.000 s/mm^2^ and f the ADC map show no restricted diffusion signs. These findings are consistent with prominent periprostatic venous plexus (*white arrows*)

**Fig. 19:**
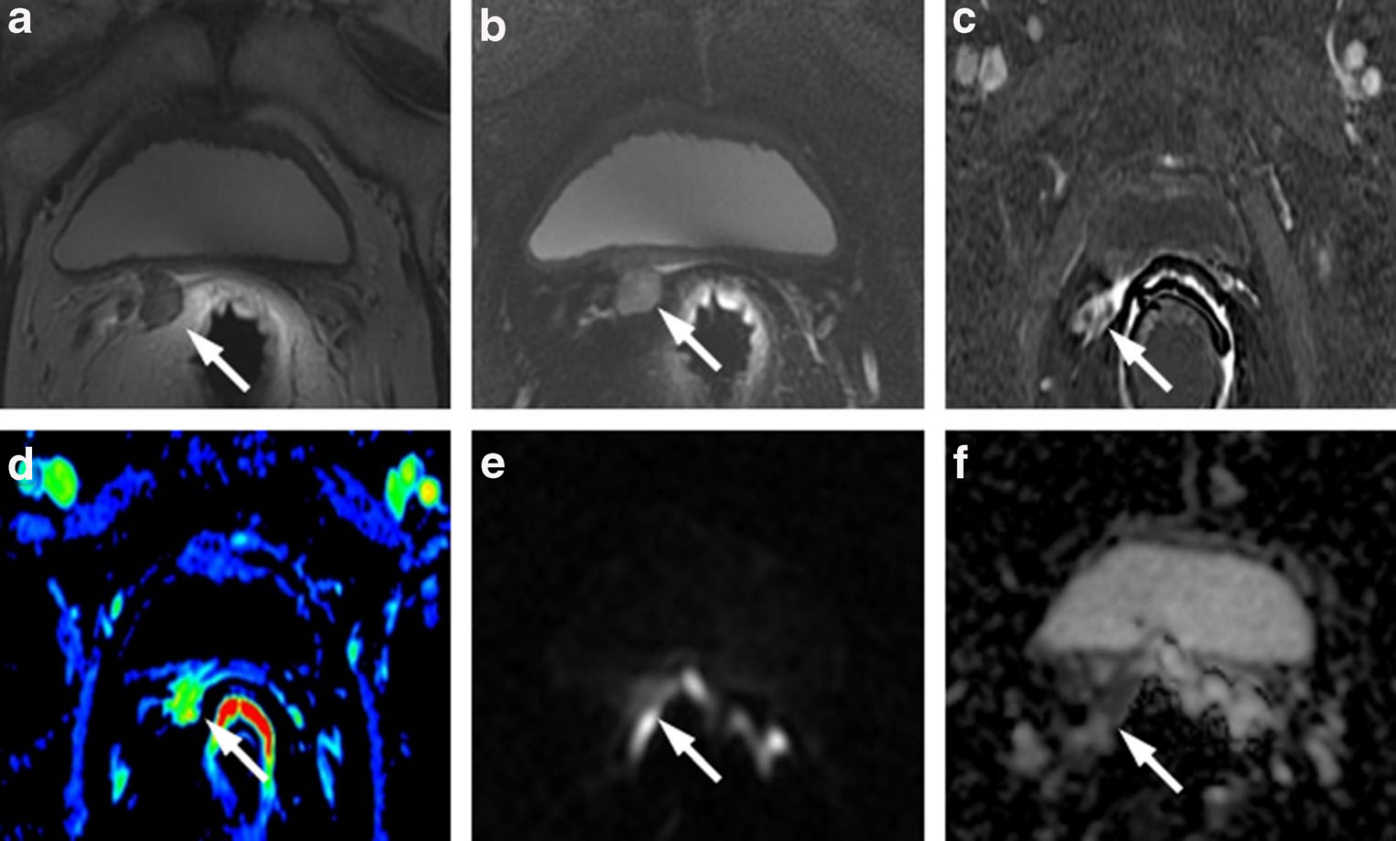
MR images of a 68-year-old man with prostate-specific antigen progression (PSA serum level 0.67 ng/mL) after radical retropubic prostatectomy, with suspected local recurrence. **a** Axial T2-weighted fast spin-echo (6567/111) and **b** axial T2-weighted fast spin-echo fat saturated (7216/118) show on the zone previously occupied by the proximal portion of right seminal vesicle, a hyperintense nodular tissue (arrow). **c** Gradient-echo T1-weighted subtracted image and **d** colour DCE map shows a well-defined area of marked enhancement (*arrow*) on the same location as the solid nodular tissue seen on T2-weighted images. **e** Diffusion weighted images with a b value of 3.000 s/mm^2^ and **f** ADC map show a focal area of restricted diffusion (*arrow*) corresponding to the solid nodular tissue detected on T2-weighted images and the enhancing zone seen on dynamic contrast-enhancing images. These findings are consistent with local recurrence (*white arrows*)

Up to now, DCE-MRI was assumed to be the most reliable MRI for detecting local prostate cancer recurrence in patients with biochemical progression after radical prostatectomy. In one study, sensitivity and specificity increased respectively from 61.4 % and 82.1 % (when using unenhanced MR imaging) to 84.1 % and 89.3 % (when using “static” MR imaging sequences acquired 20, 60, and 120 seconds after administration of intravenous contrast material) [42]. Sciarra et al. [46] reported areas under the receiver operating characteristic (ROC) curves of 0.94, 0.93, and 0.96 for MR spectroscopic imaging, dynamic contrast-enhanced MR imaging, and the combination of both, respectively, in 50 patients at high risk of local recurrence after radical prostatectomy. Panebianco et al. reported that the accuracy of DWI (in particular, using b value = 3000 s/mm^2^) is slightly lower than DCE (92 % versus 93 %) in depicting loco-regional relapse because DWI images are more affected by intrinsic distortion artefacts and background noise than DCE images, although there are some cases in which DCE is doubtful and DWI is of primary importance for local recurrence depiction [17] (Figs. 18 and 19).

### Verumontanum

**Fig. 20:**
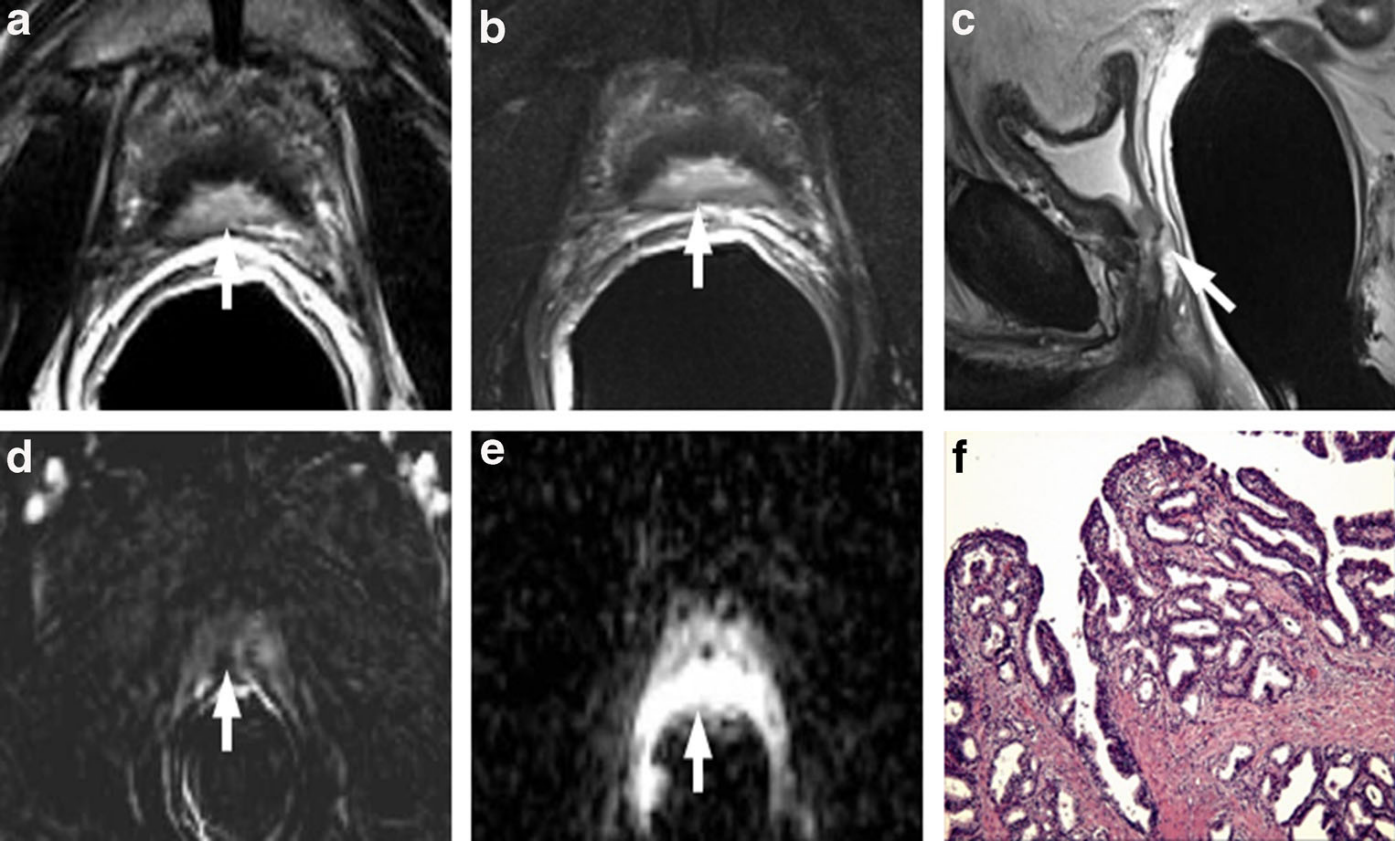
Hypertrophic verumontanum of a 66-year-old man with prostate-specific antigen progression (PSA serum level 0.75 ng/mL) after radical retropubic prostatectomy. **a** Axial T2-weighted fast spin-echo (6330/115), **b** axial T2-weighted fat saturated fast spin-echo image (8450/115) and **c** sagittal T2-weighted fast spin-echo (5000/114) show a small tissue at the bladder neck location, which is slightly hyperintense compared to pelvic muscles (*arrow*). **d** Axial Gradient-echo T1-weighted subtracted image showing no signs of enhancement of the suspicious pathological tissue detected on T2-weighted images. **e** Axial ADC map reconstructed from images obtained at b values of 0, 500 and 1000 s/mm^2^ show a bright area corresponding to the abnormal hyperintense tissue seen on T2-weighted images, which is a finding suggestive for the absence of restricted diffusion phenomena. **f** Photomicrograph from biopsy specimen showing verumontanum

The seminal colliculus or verumontanum of the prostatic urethra is a landmark near the entrance of the seminal vesicles. This microscopic recess sometimes remains in place after radical prostatectomy [47], and, when hypertrophic and inflamed, it may mimic a local recurrence [48], showing a slightly hyperintense signal intensity compared to pelvic muscles on T2-weighted images and enhancement on T1-weighted perfusion images. DWI is very useful in such a finding, because the remaining verumontaum does not show restriction diffusion phenomena (Fig. 20).

### Pitfalls after Radiation/Hormone Deprivation Therapy

The first important aspect to consider when evaluating possible recurrence after Radiation Therapy (RT) is that recurrence tends to occur at the site of the primary (pre-RT) tumour [49, 50]. The relative signal intensity difference between the treated gland and a cancerous lesion is typically less than in the untreated gland, however, often making the lesions less conspicuous [18].

**Fig. 21:**
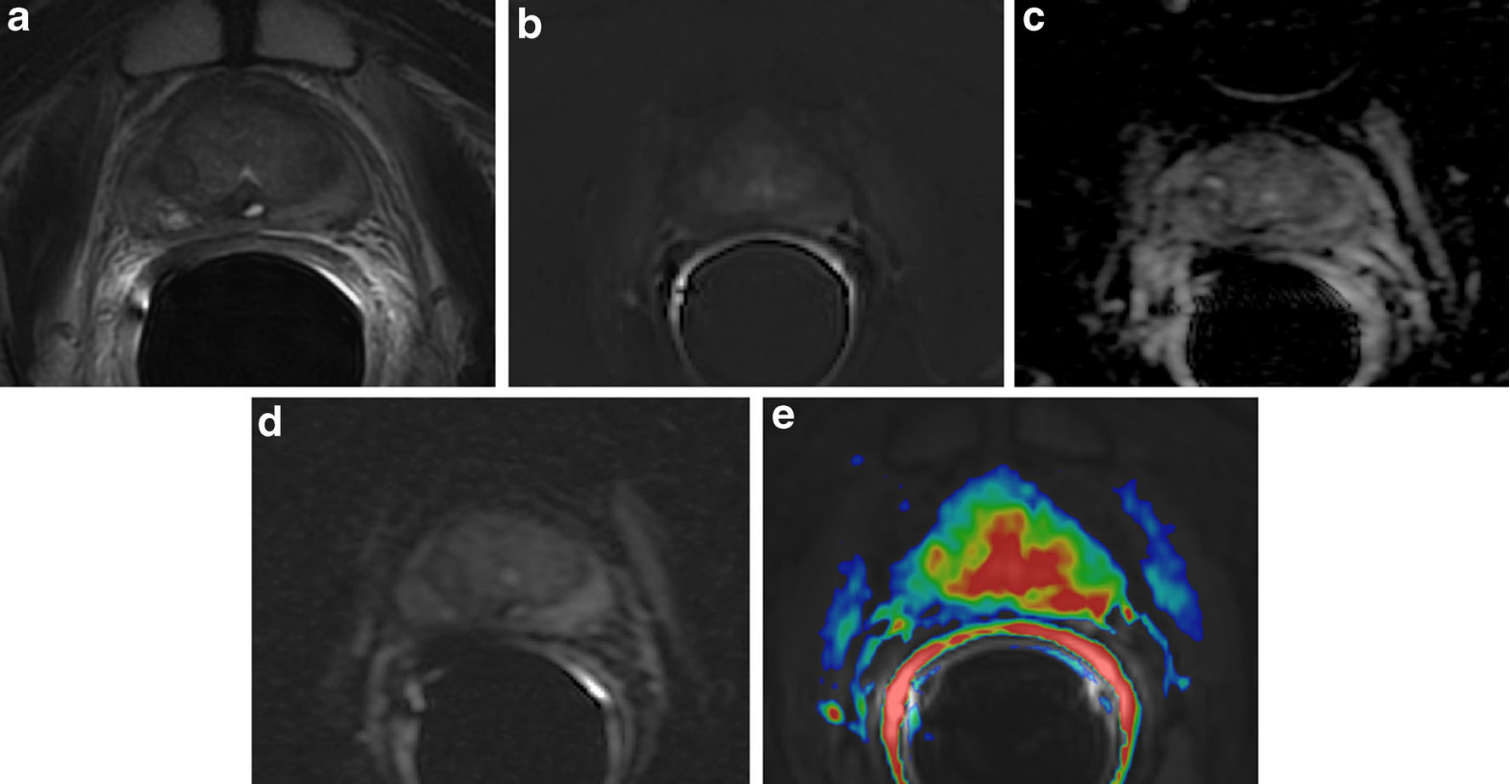
Post-RT treatment. **a** Axial T2-weighted fast spin-echo image showing an area of intermediate signal intensity in the peripheral posterior left portion of the gland after 3 months post-RT treatment suspected for recurrence. **b** Axial perfusion gradient-echo T1-weighted image shows mild enhancement in the suspected area. **c** ADC map and **d** axial DWI image with b value 1000 mm^2^/sec showing no areas of suspected restriction of water diffusivity. **e** colour DCE MR map showing marked enhancement in posterior left peripheral zone, corresponding to post-RT outcomes

A focal low signal intensity area in the peripheral zone on T2-weighted images may represent a treated tumour and not necessarily cancer recurrence, and recurrent tumours may not be apparent on T2WI [18]. In these situations, there may be a role for multiparametric assessment with MRSI, DW-MR imaging and DCE-MR imaging. In a study to evaluate prostate cancer recurrence after radiation therapy, spectroscopy was more sensitive than conventional MR imaging and digital rectal examination (77 % versus 68 % and 16 %, respectively), though it was also much less specific (78 % versus 90 % for both MR imaging and digital rectal examination) [51], probably as a result of metabolic alterations in benign tissue after RT. Dynamic contrast-enhanced MR imaging has also been reported to improve the detection of recurrence after RT by showing early arterial enhancement of tumour nodules and early washout [52] (Fig. 21). In addition, Kim et al. [53] reported that the area under the curve for predicting recurrence after RT improved from 0.61 with T2-weighted imaging to 0.88 when DW MR imaging was added to T2-weighted imaging (*p* < 0.01). In a preliminary report on the use of mp-MRI imaging in patients suspected of having recurrence after RT, Akin et al. [54] recently reported that the area under the curve for two readers increased from 0.64 and 0.53 when using T2-weighted imaging alone to 0.95 and 0.86 when using the combination of T2-weighted, DW, and dynamic contrast-enhanced MR imaging. Radiation treatment may also induce irregularities of capsular profile that may hinder the evaluation of extracapsular extension of recurrence [18]. As regards MRSI used as a tool for monitoring response to therapy, it is very important to know that a temporary peak of choline may occur in the first 3 months after radiation therapy or hormone deprivation therapy, because in the early post-treatment period, Ci concentration decreases faster than Cho and Creatine, and the ratio Cho + Cr/Ci increases > 1.5, mimicking a residual or recurrent disease [55]. Therefore, MRSI should be performed at least 3 months after radiation therapy or hormone deprivation therapy in order to avoid this kind of pitfall.

### Pitfalls after Focal Therapies

**Fig. 22:**
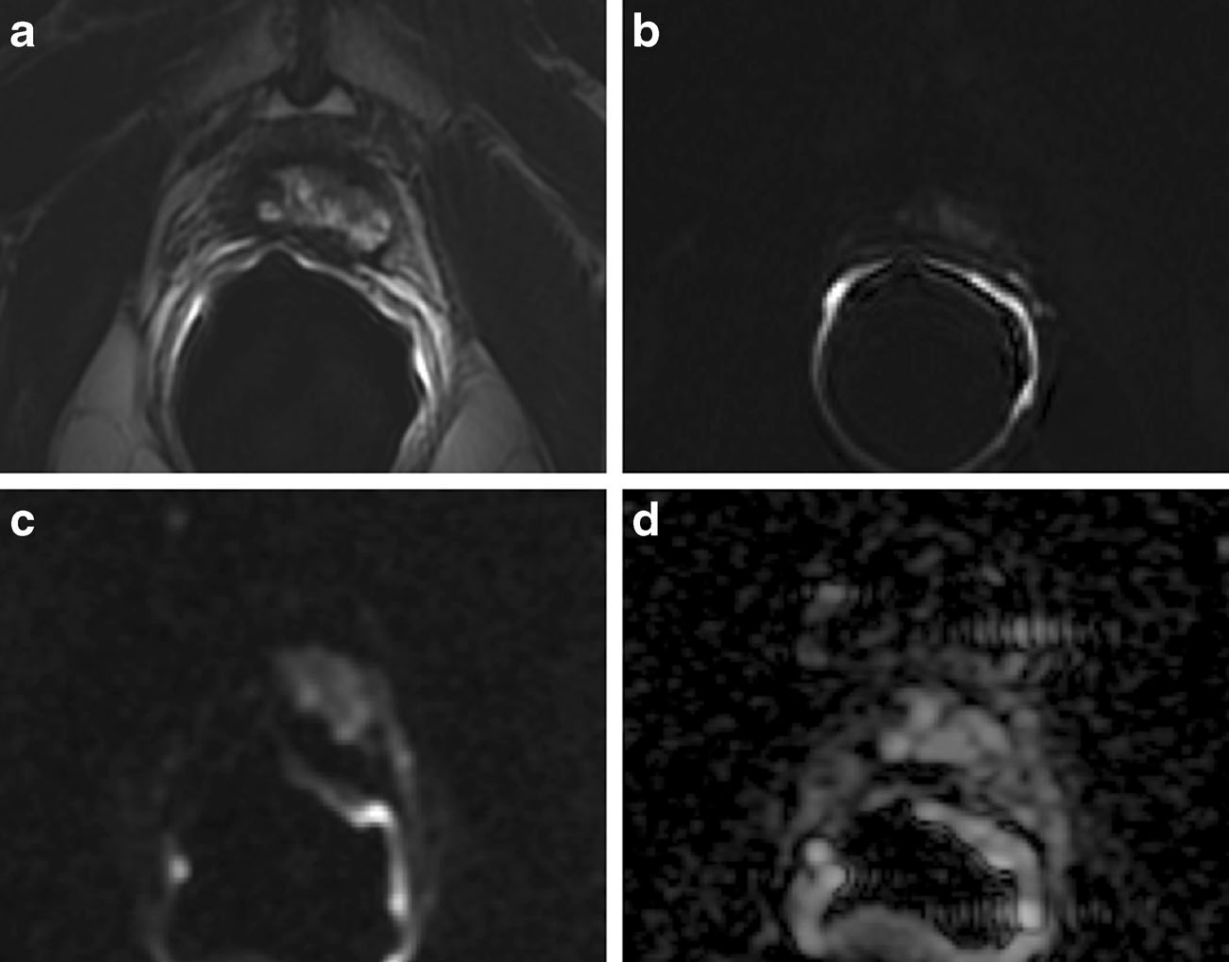
Post-HIFU treatment. **a** Axial T2-weighted fast spin-echo image showing an area of intermediate signal intensity in the anterior left portion of the gland after 3 months post-HIFU treatment, suspected for recurrence. **b** Axial perfusion gradient-echo T1-weighted image shows no significant enhancement in the suspected area. **c** Axial DWI image with b value 1000 mm^2^/sec, showing a higher hyperintense area in the anterior left portion of the prostate, **d** ADC map image showing no areas of significant restriction of water diffusivity, corresponding with post-HIFU treatment outcomes

**Fig. 23:**
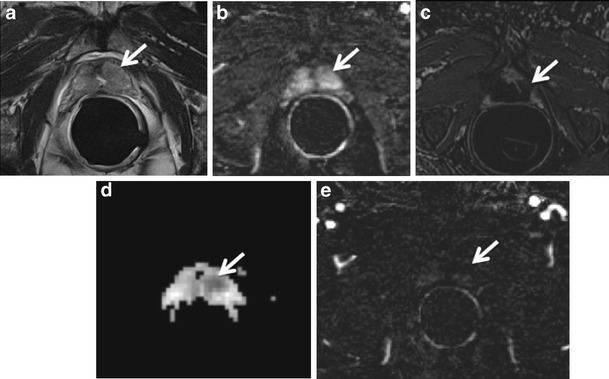
Post-MRgFUS treatment. **a** Pre-treatment axial T2-weighted fast spin-echo image and **b** post-contrast subtracted image showing a focal lesion on the postero-lateral aspect of the left third mid-gland with enhancement consistent with prostate cancer. **c** Post-treatment axial T1-weighted post-contrast subtracted image showing ablation of the area. **d** ADC map 6 months after MRgFUS treatment showing a focal zone of restricted diffusion suspected for local recurrence. **e** Post-contrast subtracted image showing no focus of contast enhancement. These findings are consistent with post-MrgFUS treatment fibrotic tissue

Currently, focal therapies, such as cryotherapy, high-intensity focused ultrasound and photodynamic therapy, are becoming a reliable and recognized form of treatment for prostate cancer as an alternative to surgery or to radiation treatment [56]. Focal treatments may be used to treat the entire prostate or to target specific prostate regions, and can be delivered by using a transrectal or transperineal approach and with US or MR guidance. Depending on the extent of the treatment, loss of zonal differentiation, thickening of the prostatic capsule, and periprostatic fibrosis and scarring may be present. In general, enhancing soft-tissue lesions after focal treatments should be considered suggestive of recurrence. Nevertheless, it may be difficult to differentiate viable tumour from reactive enhancing prostate tissue, particularly at the margins of the treated area (Figs. 22 and 23).

After cryotherapy, heterogeneous enhancement intermixed with areas of necrosis and thickening of the prostatic capsule, urethra and rectal wall are seen on T1-weighed (T1w) images [57].

After HIFU, ablation-induced changes in the region of the lesions appear on contrast-enhanced T1w images as non-enhancing hypointense regions with 3–8 mm thick peripheral rims of enhancement that resolve within 3–5 months showed that at 6 months, the prostate is of predominantly low signal intensity on T2w images and that there is a median volume reduction of 61 % [58]. They also concluded that the volume of enhancing prostate tissue on the initial image after treatment correlated well with serum PSA level nadir (Spearman’s r = 0.90, *P* < 0.001) and with volume at 6 months (Pearson’s r = 0.80, *P* = 0.001). After photodynamic therapy, MRI may be used to assess the extent and distribution of the expected necrosis in the target region. In one study [59], most patients showed marked irregularity at the treatment boundary, that was best appreciated on T1w images after i.v. administration of contrast material, with areas of enhancement (viable tissue) interposed between non-enhancing low-signal-intensity regions (necrosis) [18].

Enhancing soft tissue lesions after focal treatments should be considered suggestive of residual/recurrence, just as they are after other forms of treatment. It is important to be aware that a recurrent lesion may present in conjunction with normal post-treatment appearances. Furthermore, the characteristics typically associated with recurrence on T2w images may not represent recurrence in some cases. In some cases of recurrence, these features simply fail to appear [57]. Some authors suggest that MRSI is superior to MRI for the differentiation of cancer voxels from necrosis voxels [60], but at present, MRSI is not widely used to aid clinical decision making, and is therefore insufficient to give a conclusive statement. After HIFU, the detection of recurrent or residual disease could be hindered by diffuse or multifocal areas of low signal intensity on T2w MRI [61]. A short time to peak enhancement, early washout, and other pharmacokinetic parameters seen on DCE-MRI in patients with untreated prostate cancer can also be present in cases of recurrence after HIFU [60]. A study showed that for prediction of local tumour progression of prostate cancer after high intensity focused ultrasound, dynamic contrast-enhanced MR imaging was more sensitive but less specific than the combination of T2-weighted and DW MR imaging [62]. Despite the sensitivity of dynamic contrast-enhanced imaging for the detection of progressive tumours after ablative therapy, areas of residual benign prostatic hypertrophy may show hypervascularity, thereby yielding false-positive findings and limiting the specificity of dynamic contrast-enhanced imaging [63]. Diffusion-weighted imaging, on the other hand, may provide greater specificity, albeit lower sensitivity for detection of viable tumours after ablative therapy [64]. Therefore, suspicious lesions should always be confirmed by (targeted) biopsy.

## Artefacts and Iatrogenic Changes

### Mispositioned Endorectal Coil

**Fig. 24:**
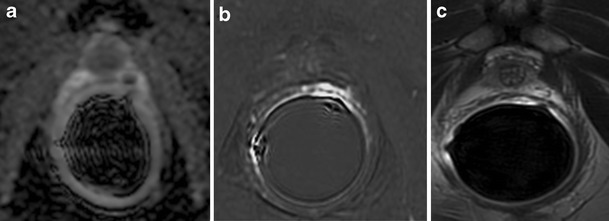
Mispositioned endorectal coil. **a** ADC map (DWI acquired with b value = 1000 mm^2^/sec and **b** 3000 mm^2^/sec) showing focal low signal intensity on the left side of the apex. **b** Gradient-echo T1-weighted subtracted image showing at the same site strong focal enhancement. According to the PIRADS v2 scoring system, the score for DWI and DCE is, respectively, 4 and +, which means highly clinically significant cancer is likely to be present. **c** Axial T2-weighted fast spin-echo image (6006/110) display no differences in signal intensity between left and right postero-lateral aspect, and it represents the decisive sequence confirming the mispositioned endorectal coil

At a standard clinical field strength of 1.5 T, an endorectal coil (ERC) is recommended for improving the signal-to-noise ratio with subsequent spatial resolution to allow reliable cancer delineation in a clinically reasonable time frame. Also, at 3 Tesla, the use of an ERC increases the signal-to-noise ratio with improved image quality and allows better localization and staging performance [11]. The ERC, however, must be correctly positioned, and, if necessary, readjusted in order to position the coil antenna in a plane perpendicular to the left-right phase encoding direction. When incorrectly positioned (e.g., it is inserted sidelong), the ERC can cause a characteristic artefact on trace DWI or high b value calculated images. This consists of a hyperintense area in the postero-lateral aspect of the peripheral zone, near to the wrongly placed antenna (Fig. 24). However, the ADC maps and T2-weighted images do not have this artefact. Thus if these images are “normal”, the pathologic finding of the trace DWI or high b value images can be excluded. The ERC must also be correctly plugged into a scanner interface. If not, signals captured by the ERC may not be transmitted for image reconstruction, leaving a hypointense curved artefact along the boundaries of the coil, increased central image noise and low signal in the prostate gland (image to be added). Patient or bowel movements during image acquisition may cause repetitive circular artefacts along the boundaries of the ERC (image to be added). They can be minimized by rectal emptying and a spasmolytic drug administered prior to the examination.

### Post-biopsy Changes

**Fig. 25:**
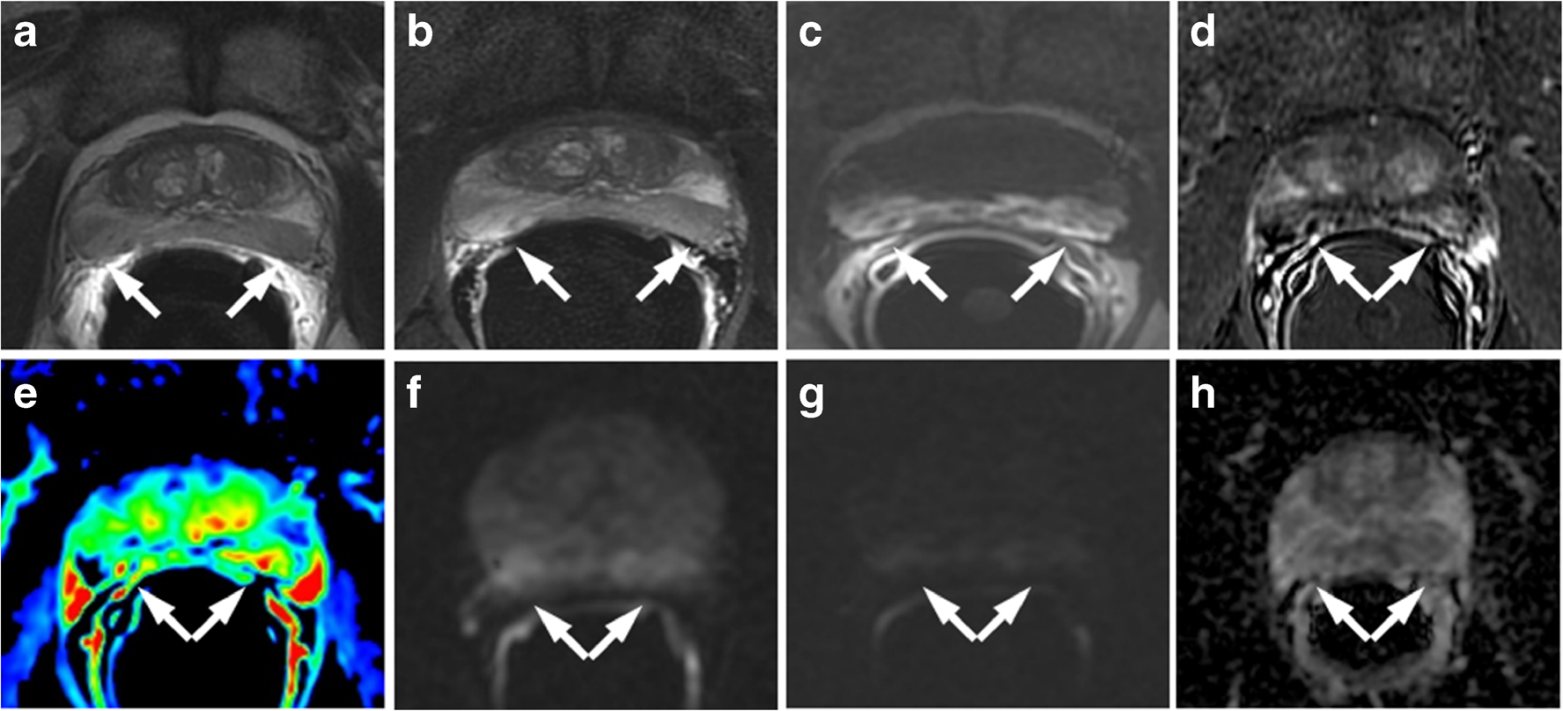
Post-biopsy haemorrhage in the midgland of a 69-year-old man with a PSA serum level of 4.7 ng/mL. **a** Axial T2-weighted fast spin-echo image (5525/109) and **b** axial fat saturated T2-weighted fast spin-echo image (5981/116) showing a large hypointense zone involving the left posterior site of the midgland and a smaller zone of low signal intensity in the contralateral side (*white arrows*). **c** The first axial perfusion gradient-echo T1-weighted image acquired before the injection of contrast medium displays diffuse high signal intensity in the midgland representing recent post-biopsy haemorrhage. **d** Gradient-echo T1-weighted subtracted image and **e** colour DCE MR map showing a real hypervascular zone in the left midgland, which appear smaller in size than in **a**, **b** and **c**. **f** Axial DWI image with b value = 1000 mm^2^/sec and **g** 3000 mm^2^/sec, and **h** ADC map showing mild diffusion restricted phenomena in the left posterior aspect of the midperipheral zone. Pathologic correlation after prostatectomy yielded prostate adenocarcinoma, with a Gleason score of 6 in left midland

Residual post-biopsy haemorrhage can lead to both false-negative and false-positive results [16]. Moreover, the damage induced by the biopsy needle may determine a transient irregularity of the periprostatic capsule, leading to an overestimation of extraprostatic tumour extension. The signal intensity (SI) of post-biopsy haemorrhage on T1 and T2-weighted images changes depending on the state of haemoglobin degradation. Mp-MRI of the prostate should ideally be indicated at least 2 months after biopsy when complete resorption of the haemorrhage is expected. The post-biopsy haemorrhage appears as a hypointense zone on T2-weighted images and shows a high SI on T1-weighted images. Because PCa appears as a low SI zone on T2-weighted with high SI on post-contrast T1-weighted images, post-biopsy haemorrhage may enclose a PCa focus and cause an overestimation of PCa extension. DW imaging may not be a very useful tool in identifying the real pathological focus, because post-biopsy haemorrhage may show restricted diffusion phenomena of water molecules with a low SI appearance on ADC maps. In this context, post-contrast subtraction imaging is mandatory to depict the suspicious focus of PCa because on T1-weighted subtraction images, the hyperintensity of the hematoma is effaced and the enhancing nodule is well depicted (Fig. 25).

### Lymphoceles

**Fig. 26:**
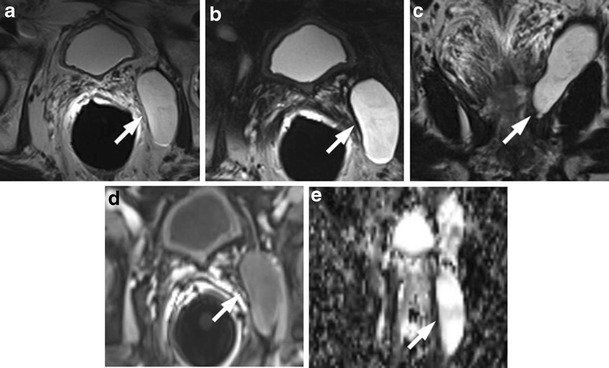
MR images of a 64-year-old man with prostate-specific antigen progression (PSA serum level 0.62 ng/mL) after radical retropubic prostatectomy, with suspected local recurrence. **a** Axial T2-weighted fast spin-echo (6200/112), **b** axial T2-weighted fat saturated fast spin-echo (8300/112) and **c** coronal T2-weighted fast spin-echo (4110/114) image showing a unilocular, thin walled hyperintense cystic-like mass at the obturator lymph nodes location. **d** Axial post-contrast T1-weighted fat saturated 3D FLASH (VIBE) image showing no signs of enhancement of the cystic mass. **e** Axial ADC map reconstructed from images obtained at b values of 0, 500 and 1000 s/mm^2^ show a bright area corresponding to the cystic mass seen on T2-weighted images. These findings are consistent with lymphocele (*white arrows*)

According to EAU guidelines, pelvic lymphadenectomy is necessary if serum PSA level is > 20 mg/dL or cT > 2b or Gleason > 7. Lymphoceles may occur at the site of lymph adenectomy and are found along the lymph node chains within the pelvis and paraaortic region. Lymphoceles are fluid filled cysts without an epithelial lining and have been reported to occur in 12–24 % of patients [65]. They usually become detectable 3–8 weeks after surgery, but may persist for up to a year. They have low signal intensity on T1-weighted images and intermediate to high signal intensity on T2-weighted images, and are typically thin-walled and unilocular and do not enhance on MR images after intravenous contrast agent administration (Fig. 26). They should not be confused with other postoperative complications such as urinoma (direct anatomic contiguity with the urinary tract), hematoma (contains blood-degradation products), abscess (thick walls, internal septations, heterogenous content, marked wall enhancement, surrounding inflammatory changes) or necrotic lymphadenopathy [18].

## References

[1] Walz J, Burnett AL, Costello AJ, Ja E, Graefen M, Guillonneau B (2010). Acritical analysis of the current knowledge of surgical anatomy related to optimization of cancer control and preservation of continence and erection incandidates for radical prostatectomy. Eur Urol.

[2] Panebianco V, Barchetti F, Sciarra A, Ciardi A, Indino EL (2015). Multiparametric magnetic resonance imaging vs. standard care in men being evaluated for prostate cancer: a randomized study. Urol Oncol.

[3] Fütterer JJ (2007). MR imaging in local staging of prostate cancer. Eur J Radiol.

[4] Hoeks CM, Barentsz JO, Hambrock T (2011). Prostate cancer: multiparametric MR imaging for detection, localization, and staging. Radiology.

[5] Colleselli D, Hennenlotter J, Schilling D (2011). Impact of clinical parameters on the diagnostic accuracy of endorectal coil MRI for the detection of prostate cancer. Urol Int.

[6] Hambrock T, Fütterer JJ, Huisman HJ (2008). Thirty two- channel coil 3T magnetic resonance-guided biopsies of prostate tumor suspicious regions identified on multimodality 3T magnetic resonance imaging: technique and feasibility. Invest Radiol.

[7] Franiel T, Lüdemann L, Rudolph B (2008). Evaluation of normal prostate tissue, chronic prostatitis, and prostate cancer by quantitative perfusion analysis using a dynamic contrast-enhanced inversion-prepared dual-contrast gradient echo sequence. Invest Radiol.

[8] Tan CH, Wei W, Johnson V (2012). Diffusion-weighted MRI in the detection of prostate cancer: meta-analysis. AJR Am J Roentgenol.

[9] Wu LM, Xu JR, Gu HY (2012). Usefulness of diffusion weighted magnetic resonance imaging in the diagnosis of prostate cancer. Acad Radiol.

[10] Morgan VA, Riches SF, Giles S (2012). Diffusion weighted MRI for locally recurrent prostate cancer after external beam radiotherapy. AJR Am J Roentgenol.

[11] Heijmink SW, Scheenen TW, Fütterer JJ (2007). Prostate and lymph node proton magnetic resonance (MR) spectroscopic imaging with external array coils at 3 T to detect recurrent prostate cancer after radiation therapy. Invest Radiol.

[12] Caivano R, Cirillo P, Balestra A (2012). Prostate cancer in magnetic resonance imaging: diagnostic utilities of spectroscopic sequences. J Med Imaging Radiat Oncol.

[13] Vilanova JC, Barceló-Vidal C, Comet J (2011). Usefulness of prebiopsy multifunctional and morphologic MRI combined with free-to-total prostate-specific antigen ratio in the detection of prostate cancer. AJR Am J Roentgenol.

[14] Rischke HC, Schäfer AO, Nestle U (2012). Detection of local recurrent prostate cancer after radical prostatectomy in terms of salvage radiotherapy using dynamic contrast enhanced-MRI without endorectal coil. Radiat Oncol.

[15] Chen YJ, Chu WC, Pu YS (2012). Washout gradient in dynamic contrast-enhanced MRI is associated with tumor aggressiveness of prostate cancer. J Magn Reson Imaging.

[16] Verma S, Turkbey B, Muradyan N (2012). Overview of dynamic contrast-enhanced MRI in prostate cancer diagnosis and management. AJR Am J Roentgenol.

[17] Panebianco V, Barchetti F, Sciarra A, Musio D, Forte V, Gentile V (2013). Prostate cancer recurrence after radical prostatectomy: the role of 3-T diffusion imaging in multi-parametric magnetic resonance imaging. Eur Radiol.

[18] Vargas HA, Wassberg C, Akin O, Hricak H (2012). MR imaging of treated prostate cancer. Radiology.

[19] Liberman L, Menell JH (2002). Breast imaging reporting and data system (BI-RADS). Radiol Clin North Am.

[20] Barentsz JO, Richenberg J, Clements R (2012). ESUR prostate MR guidelines 2012. Eur Radiol.

[21] Rosenkrantz AB, Taneja SS (2014). Radiologist, be aware: ten pitfalls that confound the interpretation of multiparametric prostate MRI. AJR Am J Roentgenol.

[22] Heijmink SW, Fütterer JJ, Hambrock T (2007). Prostate cancer: body-array versus endorectal coil MR imaging at 3 T-- comparison of image quality, localization, and staging performance. Radiology.

[23] Oto A, Kayhan A, Jiang Y (2010). Prostate cancer: differentiation of central gland cancer from benign prostatic hyperplasia by using diffusion-weighted and dynamic contrast-enhanced MR imaging. Radiology.

[24] McNeal JE, Redwine EA, Freiha FS, Stamey TA (1988). Zonal distribution of prostatic adenocarcinoma. Correlation with histologic pattern and direction of spread. Am J Surg Pathol.

[25] Greene DR, Wheeler TM, Egawa S (1991). A comparison of the morphological features of cancer arising in the transition zone and in the peripheral zone of the prostate. J Urol.

[26] Shannon BA, McNeal JE, Cohen RJ (2003). Transition zone carcinoma of the prostate gland: a common indolent tumour type that occasionally manifests aggressive behaviour. Pathology.

[27] Akin O, Sala E, Moskowitz CS (2006). Transition zone prostate cancers: features, detection, localization, and staging at endorectal MR imaging. Radiology.

[28] Kim CK, Park BK, Han JJ (2007). Diffusion-weighted imaging of the prostate at 3 T for differentiation of malignant and benign tissue in transition and peripheral zones: preliminary results. J Comput Assist Tomogr.

[29] Tamada T, Sone T, Jo Y (2008). Apparent diffusion coefficient values in peripheral and transition zones of the prostate: comparison between normal and malignant prostatic tissues and correlation with histologic grade. J Magn Reson Imaging.

[30] Kim CK, Park BK, Lee HM, Kwon GY (2007). Value of diffusion-weighted imaging for the prediction of prostate cancer location at 3T using a phased-array coil: preliminary results. Invest Radiol.

[31] Sato C, Naganawa S, Nakamura T (2005). Differentiation of noncancerous tissue and cancer lesions by apparent diffusion coefficient values in transition and peripheral zones of the prostate. J Magn Reson Imaging.

[32] Hoeks CM, Hambrock T, Yakar D, de Kaa CA H, Feuth T, Witjes JA (2013). Transition zone prostate cancer: detection and localization with 3-T multiparametric MR imaging. Radiology.

[33] Bour L, Schull A, Delongchamps NB, Beuvon F, Muradyan N, Legmann P (2013). Multiparametric MRI features of granulomatous prostatitis and tubercular prostate abscess. Diagn Interv Imaging.

[34] Bouyé S, Potiron E, Puech P, Leroy X, Lemaitre L, Villers A (2009). Transition zone and anterior stromal prostate cancers: zone of origin and intraprostatic patterns of spread at histopathology. Prostate.

[35] Jager GJ, Barentsz JO, Oosterhof GO, Witjes JA, Ruijs SJ (1996). Pelvic adenopathy in prostatic and urinary bladder carcinoma: MR imaging with a three-dimensional T1- weighted magnetization-prepared-rapid gradient- echo sequence. AJR Am J Roentgenol.

[36] Harisinghani MG, Barentsz J, Hahn PF, Deserno WM, Tabatabaei S, van de Kaa CH (2003). Noninvasive detection of clinically occult lymph-node metastases in prostate cancer. N Engl J Med.

[37] Young HH (1905). VIII. Conservative perineal prostatectomy: the results of two years’ experience and report of seventy-five cases. Ann Surg.

[38] Cooperberg MR, Lubeck DP, Meng MV, Mehta SS, Carroll PR (2004). The changing face of low-risk prostate cancer: trends in clinical presentation and primary management. J Clin Oncol.

[39] Bianco FJ, Scardino PT, Eastham JA (2005). Radical prostatectomy: long-term cancer control and recovery of sexual and urinary function (“trifecta”). Urology.

[40] Eastham JA, Scardino PT, Kattan MW (2008). Predicting an optimal outcome after radical prostatectomy: the trifecta nomogram. J Urol.

[41] Patel VR, Coelho RF, Chauhan S (2010). Continence, potency and oncological outcomes after robotic-assisted radical prostatectomy: early trifecta results of a high-volume surgeon. BJU Int.

[42] Cirillo S, Petracchini M, Scotti L (2009). Endorectal magnetic resonance imaging at 1.5 Tesla to assess local recurrence following radical prostatectomy using T2-weighted and contrast-enhanced imaging. Eur Radiol.

[43] Sella T, Schwartz LH, Swindle PW (2004). Suspected local recurrence after radical prostatectomy: endorectal coil MR imaging. Radiology.

[44] Sella T, Schwartz LH, Hricak H (2006). Retained seminal vesicles after radical prostatectomy: frequency, MRI characteristics, and clinical relevance. AJR Am J Roentgenol.

[45] Hricak H, Carrington BM (1991). MRI of the pelvis: a text atlas.

[46] Sciarra A, Panebianco V, Salciccia S (2008). Role of dynamic contrast-enhanced magnetic resonance (MR) imaging and proton MR spectroscopic imaging in the detection of local recurrence after radical prostatectomy for prostate cancer. Eur Urol.

[47] Sfoungaristos S, Kontogiannis S, Perimenis P (2013). Early continence recovery after preservation of maximal urethral length until the level of verumontanum during radical prostatectomy: primary oncological and functional outcomes after 1 year of follow-up. Biomed Res Int.

[48] Muezzinoglu B, Erdamar S, Chakraborty S, Wheeler TM (2001). Verumontanum mucosal gland hyperplasia is associated with atypical adenomatous hyperplasia of the prostate. Arch Pathol Lab Med.

[49] Cellini N, Morganti AG, Mattiucci GC (2002). Analysis of intraprostatic failures in patients treated with hormonal therapy and radiotherapy: implications for conformal therapy planning. Int J Radiat Oncol Biol Phys.

[50] Pucar D, Hricak H, Shukla-Dave A (2007). Clinically signifi cant prostate cancer local recurrence after radiation therapy occurs at the site of primary tumor: magnetic resonance imaging and step-section pathology evidence. Int J Radiat Oncol Biol Phys.

[51] Pucar D, Shukla-Dave A, Hricak H (2005). Prostate cancer: correlation of MR imaging and MR spectroscopy with pathologic findings after radiation therapy-initial experience. Radiology.

[52] Rouvière O, Valette O, Grivolat S (2004). Recurrent prostate cancer after external beam radiotherapy: value of contrast-enhanced dynamic MRI in localizing intraprostatic tumor—correlation with biopsy findings. Urology.

[53] Kim CK, Park BK, Lee HM (2009). Prediction of locally recurrent prostate cancer after radiation therapy: incremental value of 3T diffusion-weighted MRI. J Magn Reson Imaging.

[54] Akin O, Gultekin DH, Vargas HA (2011). Incremental value of diffusion weighted and dynamic contrast enhanced MRI in the detection of locally recurrent prostate cancer after radiation treatment: preliminary results. Eur Radiol.

[55] Mueller-Lisse UG, Swanson MG, Vigneron DB (2001). Time-Dependent Effects of Hormone-Deprivation Therapy on Prostate Metabolism as Detected by Combined Magnetic Resonance Imaging and 3D Magnetic Resonance Spectroscopic Imaging. Magn Reson Med.

[56] Muller BG, Fütterer JJ, Gupta RT, Katz A, Kirkham A, Kurhanewicz J (2014). The role of magnetic resonance imaging (MRI) in focal therapy for prostate cancer: recommendations from a consensus panel. BJU Int.

[57] Kalbhen CL, Hricak H, Shinohara K (1996). Prostate carcinoma: MR imaging findings after cryosurgery. Radiology.

[58] Kirkham AP, Emberton M, Hoh IM, Illing RO, Freeman AA, Allen C (2008). MR imaging of prostate after treatment with high-intensity focused ultrasound. Radiology.

[59] Haider MA, Davidson SR, Kale AV (2007). Prostate gland: MR imaging appearance after vascular targeted photodynamic therapy with palladium-bacteriopheophorbide. Radiology.

[60] Parivar F, Hricak H, Shinohara K (1996). Detection of locally recurrent prostate cancer after cryosurgery: evaluation by transrectal ultrasound, magnetic resonance imaging, and threedimensional proton magnetic resonance spectroscopy. Urology.

[61] Rouvière O, Lyonnet D, Raudrant A (2001). MRI appearance of prostate following transrectal HIFU ablation of localized cancer. Eur Urol.

[62] Kim CK, Park BK, Lee HM, Kim SS, Kim E (2008). MRI techniques for prediction of local tumor progression after high-intensity focused ultrasonic ablation of prostate cancer. AJR Am J Roentgenol.

[63] Rosenkrantz AB, Scionti SM, Mendrinos S, Taneja SS (2011). Role of MRI in minimally invasive focal ablative therapy for prostate cancer. AJR Am J Roentgenol.

[64] Kim CK, Park BK, Lee HM, Kim SS, Kim E (2008). MRI techniques for prediction of local tumor progression after high-intensity focused ultrasonic ablation of prostate cancer. AJR.

[65] Yang DM, Jung DH, Kim H (2004). Retroperitoneal cystic masses: CT, clinical, and pathologic findings and literature review. RadioGraphics.

